# Exploratory associations of composite inflammatory-immune markers with 28-day and 1-year all-cause mortality in patients with sepsis: a retrospective cohort study

**DOI:** 10.3389/fendo.2026.1946659

**Published:** 2026-10-28

**Authors:** Jiakai Wang, Yaqi Wang, Lei Li, Chenxin Zhang, Kexin Wen, Xiaohong Pi, Kaitian Yao, Quansheng Du

**Affiliations:** 1 Graduate School, Hebei Medical University, Shijiazhuang, Hebei, China; 2 Department of Critical Care Medicine, Hebei General Hospital, Hebei Medical University, Shijiazhuang, Hebei, China; 3 Graduate School, Hebei North University, Zhangjiakou, Hebei, China; 4 Department of Emergency Medicine and Critical Care Medicine (Ward 2), Hebei General Hospital, Shijiazhuang, Hebei, China; 5 Hebei Key Laboratory of Clinical Pharmacy, Shijiazhuang, Hebei, China

**Keywords:** inflammatory-immune markers, mortality, restricted cubic spline, sepsis, subgroup analysis

## Abstract

**Aims:**

Inflammation and immune dysregulation are central to sepsis pathobiology, but the prognostic value of a broad panel of composite inflammatory-immune indices remains incompletely defined. We evaluated the associations of 18 composite inflammatory-immune markers with 28-day and 1-year all-cause mortality in sepsis patients.

**Methods:**

This single-center retrospective cohort study included 396 adult patients admitted to the intensive care unit (ICU) with sepsis. Eighteen inflammatory-immune indices were calculated from blood tests obtained within the first 24 h after ICU admission. Per-1-standard deviation (SD) multivariable Cox models were used to evaluate each marker’s association with 28-day and 1-year all-cause mortality. Subgroup analyses were performed by age (<75 vs. ≥75 years), sex, and sepsis-associated acute kidney injury status, with multiplicative interaction terms. Restricted cubic spline (RCS) analyses were used to assess nonlinearity.

**Results:**

Among 396 patients, 174 (43.9%) died within 28 days and 230 (58.1%) died within 1 year. In the primary per-1-SD multivariable Cox analyses, higher C-reactive protein-to-lymphocyte ratio (CLR) and platelet-to-lymphocyte ratio (PLR) were associated with higher 28-day mortality (CLR: HR 1.313, 95% CI 1.145–1.505; P<0.001; PLR: HR 1.253, 95% CI 1.114–1.410; P<0.001) and 1-year mortality (CLR: HR 1.258, 95% CI 1.104–1.435; P<0.001; PLR: HR 1.193, 95% CI 1.071–1.329; P = 0.001). Higher lymphocyte-to-HDL-C ratio (LHR) was inversely associated with both endpoints (28-day: HR 0.759, 95% CI 0.626–0.920; P = 0.005; 1-year: HR 0.819, 95% CI 0.703–0.954; P = 0.010). Other markers showed endpoint-dependent or otherwise variable associations. Most formal interaction tests did not indicate heterogeneity by age, sex, or sepsis-associated acute kidney injury (SA-AKI) status; nominal interactions were observed for pan-immune-inflammation value with age for both endpoints and with SA-AKI status for 1-year mortality. RCS analyses showed nonlinear associations of log_10_-transformed PLR and CLR with both endpoints and an approximately linear inverse association of log_10_-transformed LHR with 28-day mortality.

**Conclusions:**

Higher CLR and PLR and lower LHR were independently associated with both short- and long-term mortality in patients with sepsis. However, the findings were derived from a single-center retrospective cohort without independent validation and do not establish clinical prediction utility. Further multicenter studies are required to validate these markers before they can be considered for clinical risk stratification.

## 1 Introduction

Sepsis is a life-threatening organ dysfunction caused by a dysregulated host response to infection and remains a leading cause of morbidity and mortality in intensive care units worldwide (1, 2). Despite advances in antimicrobial therapy, organ support, and hemodynamic management, sepsis still accounts for an estimated 11 million deaths annually, with mortality rates varying widely depending on illness severity, comorbidity burden, pathogen type, and access to critical care (3–5). Patients who survive the acute phase often face prolonged hospitalization, increased risk of secondary infections, and substantial long-term physical and cognitive impairment, underscoring the need for accurate early risk stratification (6, 7). Acute kidney injury (AKI) is one of the most frequent complications of sepsis and is associated with higher mortality. Sepsis-associated AKI (SA-AKI) occurs in approximately 40–50% of septic patients and may reflect uncontrolled systemic inflammation and microvascular dysfunction (8–12). In recent years, accumulating evidence has highlighted the central role of immune dysregulation in sepsis. During the early phase, an exaggerated innate immune response characterized by neutrophil activation, monocyte recruitment, and release of pro-inflammatory cytokines contributes to endothelial injury and organ damage (13, 14). Simultaneously, apoptotic loss of lymphocytes and functional exhaustion of T cells drive a compensatory anti-inflammatory response that increases susceptibility to secondary infections and late mortality (15, 16). This inflammatory-immune imbalance can be captured, at least in part, by routine hematological and biochemical parameters.

A growing number of composite inflammatory-immune markers have been proposed as simple, inexpensive prognostic tools in critical illness (17, 18). These indices integrate different cellular and humoral components of the immune response and have shown prognostic value in cancer, cardiovascular disease, and infection (19–21). In sepsis, markers such as the neutrophil-to-lymphocyte ratio (NLR), platelet-to-lymphocyte ratio (PLR), systemic immune-inflammation index (SII), and systemic inflammation response index (SIRI) have been associated with disease severity and mortality (19, 22–25). More recently, indices combining inflammation with nutritional or metabolic status—such as the C-reactive protein (CRP)-to-albumin ratio (CAR), CRP-to-lymphocyte ratio (CLR), and hemoglobin-albumin-lymphocyte-platelet (HALP) score—have gained attention for their ability to reflect both immune activation and host reserve (26–28). However, most previous studies have examined only a limited number of markers, often in general septic populations, and have not systematically compared a broad panel within the same cohort or examined both short and long term outcomes (17, 18).

Despite these advances, several knowledge gaps remain. First, few studies have directly compared the prognostic performance of cellular indices such as NLR, SII, and pan-immune-inflammation value (PIV) with inflammation-nutrition indices such as CAR, CRP-albumin-lymphocyte index (CALLY), and HALP within the same sepsis cohort. Second, the generalizability of findings to high-risk subgroups of sepsis patients remains uncertain. Third, most prior investigations have focused on short-term mortality, leaving the long-term prognostic value of these markers unclear. Fourth, the multiple-testing burden inherent in comparing many indices has rarely been addressed formally, and whether any index adds discriminative value beyond established severity scores is unknown. Whether these readily available indices are independently associated with mortality and whether they provide incremental prognostic information beyond established severity scores remain uncertain. Addressing these gaps may help prioritize candidate markers for future prospective and external validation. Host-response assessment in sepsis extends beyond routine composite indices to cellular immune phenotyping (e.g., monocyte HLA-DR expression), hematological parameters (e.g., monocyte distribution width), biophysical leukocyte-response approaches (e.g., the Cytovale IntelliSep system), host gene-expression platforms (e.g., SeptiCyte RAPID and Inflammatix), protein-based host-response assays (e.g., MeMed BV), and ex vivo functional assays (e.g., LPS-induced TNF-α release). Routine blood-cell and biochemical indices are more widely accessible and can be viewed as low-cost adjuncts to these approaches (29). Accordingly, this retrospective cohort study was conducted as an exploratory, hypothesis-generating analysis to compare the associations of 18 composite inflammatory–immune markers with 28-day and 1-year all-cause mortality in patients with sepsis. The main analytical framework comprised per-1-standard deviation (SD) multivariable Cox analyses, formal subgroup interaction analyses, and restricted cubic spline (RCS) assessment of exposure-response shape. Alternative parameterizations and Benjamini-Hochberg false-discovery-rate (FDR) correction were used as sensitivity analyses to assess robustness. We additionally assessed whether candidate inflammatory-immune markers provided incremental prognostic information beyond conventional severity scores using nested-model comparisons, time-dependent discrimination measures, and reclassification metrics.

## 2 Methods

### 2.1 Study design and population

This single-center retrospective cohort study was conducted at Hebei General Hospital, a tertiary-care teaching hospital in Shijiazhuang, China. Consecutive adult patients (≥ 18 years) admitted to the ICU with a diagnosis of sepsis between January 1, 2021 and December 31, 2024 were screened for inclusion. Sepsis was defined according to the Sepsis-3 criteria as life-threatening organ dysfunction caused by a dysregulated host response to infection, quantified by an acute increase in the Sequential Organ Failure Assessment (SOFA) score of 2 points or more (1). Patients with septic shock were identified by the concurrent need for vasopressor therapy to maintain a mean arterial pressure (MAP) of at least 65 mmHg and a serum lactate concentration greater than 2 mmol/L despite adequate fluid resuscitation (30). SA-AKI was defined according to the 28th Acute Disease Quality Initiative consensus as KDIGO-defined AKI occurring within 7 days after sepsis onset, with sepsis diagnosed according to the Sepsis-3 criteria (31, 32).

Patients were excluded if they had pre-existing end-stage renal disease receiving maintenance dialysis, kidney transplantation, ICU stay less than 24 h, missing survival data, or incomplete laboratory data precluding calculation of the inflammatory-immune indices. The study was approved by the Ethics Committee of the Hebei General Hospital. The research protocol complied with the Declaration of Helsinki. Given the retrospective design and use of anonymized data, the requirement for informed consent was waived.

### 2.2 Data collection

Demographic characteristics, comorbidities, acute organ complications, vital signs, laboratory parameters, illness-severity scores, and organ-support treatments were extracted from the electronic medical record system. Unless otherwise specified, biomarker components, vital signs, laboratory measurements, and severity scores were obtained or calculated using data from the first 24 h after ICU admission. Demographic variables included age and sex. Pre-existing comorbidities included chronic heart failure (CHF), cerebrovascular disease, and diabetes, while acute organ complications included SA-AKI and acute respiratory distress syndrome (ARDS).

CHF was considered present when a pre-existing physician diagnosis was documented in the medical record. Heart failure was defined as a clinical syndrome characterized by symptoms and/or signs caused by a structural or functional cardiac abnormality and corroborated by elevated natriuretic peptide concentrations and/or objective evidence of pulmonary or systemic congestion (33). Cerebrovascular disease was defined as a documented history of cerebral infarction or cerebral hemorrhage. Diabetes was considered present when a pre-existing diagnosis was documented or when the patient was receiving insulin or oral glucose-lowering medication before ICU admission; isolated stress hyperglycemia during the index hospitalization was not sufficient for classification as diabetes (34). ARDS was diagnosed according to the Berlin definition, requiring onset within 1 week of a known clinical insult or new or worsening respiratory symptoms, bilateral pulmonary opacities not fully explained by pleural effusion, lobar or lung collapse, or pulmonary nodules, respiratory failure not fully explained by cardiac failure or fluid overload, and a ratio of arterial oxygen partial pressure to inspired oxygen fraction (PaO_2_/FiO_2_) ≤300 mmHg with positive end-expiratory pressure or continuous positive airway pressure ≥5 cmH_2_O (35).

Vital-sign and physiological variables included systolic blood pressure, diastolic blood pressure (DBP), heart rate (HR), MAP, arterial oxygen saturation (SaO_2_), and cumulative urine output during the first 24 h after ICU admission. Arterial blood gas measurements included the partial pressure of oxygen (PaO_2_), partial pressure of carbon dioxide (PaCO_2_), pH, and lactate. Core laboratory variables used in the main analyses included white blood cell (WBC) count, neutrophil count, lymphocyte count, monocyte count, hemoglobin, platelet count, creatinine, CRP, procalcitonin (PCT), high-density lipoprotein cholesterol (HDL-C), total bilirubin, and albumin. Serum calcium, uric acid, triglycerides, total cholesterol, low-density lipoprotein cholesterol (LDL-C), B-type natriuretic peptide (BNP), troponin T, alanine aminotransferase (ALT), prothrombin time, D-dimer are reported in **Supplementary Table S1**. Unless otherwise specified, the first available laboratory result obtained within 24 h after ICU admission was used.

Illness severity was evaluated using the SOFA score, Acute Physiology and Chronic Health Evaluation II (APACHE II) score, Glasgow Coma Scale (GCS), Simplified Acute Physiology Score II (SAPS II), Systemic Inflammatory Response Syndrome (SIRS) score, and Oxford Acute Severity of Illness Score (OASIS), all assessed within the first 24 h after ICU admission. Organ-support variables, including mechanical ventilation and continuous renal replacement therapy (CRRT) were ascertained during the first 48 h after ICU admission. This 48-h window was selected after review of the clinical records because it provided more complete and stable ascertainment of established early organ-support requirements than the first 24 h alone. These variables were used as adjustment covariates.

### 2.3 Definitions of inflammatory-immune markers

Eighteen inflammatory-immune markers were calculated from routine blood tests obtained within 24 h of ICU admission. The formulas were as follows:

1. SII = platelet count × neutrophil count/lymphocyte count (20);
2. NLR = neutrophil count/lymphocyte count (36);
3. Derived neutrophil-to-lymphocyte ratio (dNLR) = neutrophil count/(WBC count − neutrophil count) (37);
4. Monocyte-to-lymphocyte ratio (MLR) = monocyte count/lymphocyte count (38);
5. Neutrophil-plus-monocyte-to-lymphocyte ratio (NMLR) = (neutrophil count + monocyte count)/lymphocyte count (39);
6. SIRI = neutrophil count × monocyte count/lymphocyte count (40);
7. PIV = neutrophil count × platelet count × monocyte count/lymphocyte count (41);
8. PLR = platelet count/lymphocyte count (42);
9. Neutrophil-to-platelet ratio (NPR) = neutrophil count/platelet count (43);
10. Platelet-to-albumin ratio (PAR) = platelet count/albumin (44);
11. CAR = CRP/albumin (45);
12. CLR = CRP/lymphocyte count (46);
13. CALLY = albumin × lymphocyte count/(CRP×10) (47);
14. HALP = hemoglobin × albumin × lymphocyte count/platelet count (48);
15. Neutrophil-to-HDL-C ratio (NHR) = neutrophil count/HDL-C (49);
16. Monocyte-to-HDL-C ratio (MHR) = monocyte count/HDL-C (21);
17. Lymphocyte-to-HDL-C ratio (LHR) = lymphocyte count/HDL-C (50);
18. Platelet-to-HDL-C ratio (PHR) = platelet count/HDL-C (51);

For each marker, the standardized value was calculated using the following formula: Standardized value = (raw value-cohort mean)/cohort SD. Accordingly, a one-unit increase in the standardized value represented a one-SD increase in the corresponding original marker. Pre-specified primary parameterization: per-1-SD standardized continuous variable. Sensitivity parameterizations: untransformed continuous variable, log_10_-transformed continuous variable when appropriate, and categorical variable dichotomized at the cohort median. Median cut-offs reported in **Supplementary Table S2**.

### 2.4 Outcomes

The primary outcomes were 28-day and 1-year all-cause mortality. All-cause mortality was defined as death from any cause during the follow-up period. The 28-day all-cause mortality was calculated as the proportion of patients who died within 28 days from ICU admission, and the 1-year all-cause mortality was calculated as the proportion of patients who died within 1 year from ICU admission. To ensure that the 24-h biomarker measurement window was completed before patients entered the risk set, follow-up for the primary survival analyses began at the 24-h landmark and ended at death, loss to follow-up, or the relevant administrative endpoint, whichever occurred first. Survival status was determined from the electronic medical record system, and telephone follow-ups were conducted when necessary to confirm the vital status. Among the 396 patients in the final analytical cohort, 1-year vital status was available for all participants.

### 2.5 Statistical analysis

Missingness was assessed for all variables included in the analyses, and the missingness rate was less than 5% for every variable. All analyses were therefore conducted using a complete-case approach, and multiple imputation was not performed. A total of 478 patients were screened for eligibility. Five patients aged <18 years, 18 patients with an ICU stay <24 h or death before the 24-h landmark, 12 patients receiving maintenance dialysis or with a history of kidney transplantation, 22 patients with unavailable survival data, and 25 patients with insufficient laboratory data were excluded. The final complete-case cohort therefore comprised 396 patients, as shown in **Figure 1**.

**Figure 1:**
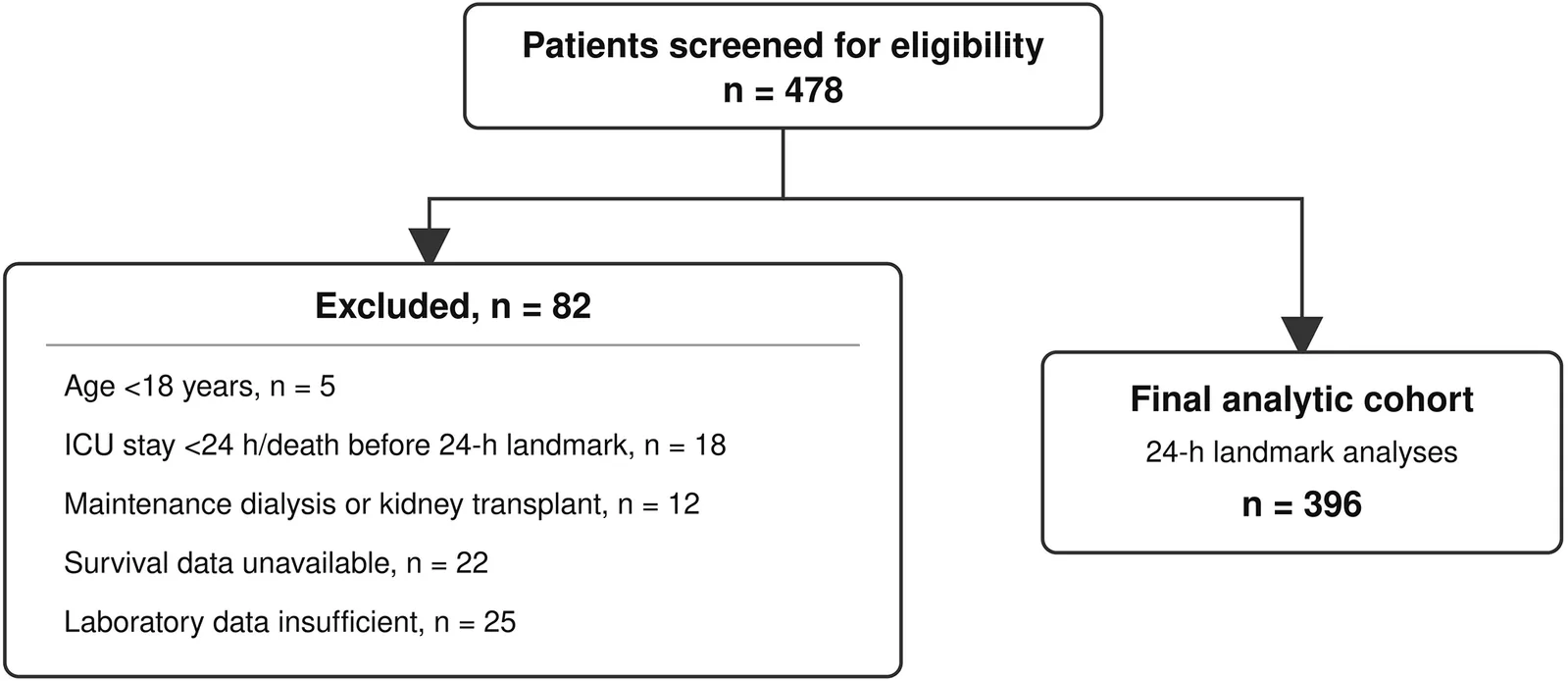
Flow diagram of participant selection.

Continuous variables were assessed for normality using the Shapiro-Wilk test and expressed as median with interquartile range; categorical variables were expressed as counts with percentages. Group comparisons were performed using the Mann-Whitney U test for continuous variables and the chi-square test or Fisher’s exact test for categorical variables, as appropriate.

Each inflammatory-immune marker was analyzed per unit, per 1-SD increase in the standardized continuous value, after log_10_ transformation, and as a categorical variable dichotomized at the cohort median. The prespecified primary parameterization was the per-1-SD standardized value. Univariate Cox proportional hazards regression was first performed to evaluate associations with 28-day and 1-year all-cause mortality. Variables with P < 0.05 in univariate analysis, together with clinically important variables identified *a priori* by the study team, were selected to construct multivariate Cox regression models. These multivariate models were used to further evaluate the significant associations of these inflammatory-immune markers with 28-day and 1-year all-cause mortality risk. For 28-day mortality, the final model included age, HR, MAP, 24-h urine output, SaO_2_, PaCO_2_, pH, lactate, serum potassium, PCT, BNP, ALT, prothrombin time, D-dimer, SOFA score, APACHE II score, GCS, SAPS II score, SIRS score, OASIS score, mechanical ventilation within 48 h, and CRRT within 48 h. For 1-year mortality, the final model included age, 24h urine output, PaCO_2_, SaO_2_, CRP, PCT, lactate, serum potassium, ALT, BNP, SOFA score, APACHE II score, GCS, SAPS II score, OASIS score, and mechanical ventilation within 48 h. CRP was excluded from models evaluating CAR, CLR, and CALLY because it is a mathematical component of these indices. The 48-h organ-support window was used because it yielded more complete and stable ascertainment of early support requirements than a 24-h window. To evaluate whether adjustment for organ-support variables assessed after the 24-h landmark influenced the results, all 36 primary standardized marker-outcome Cox models were refitted after excluding the 48-h organ-support covariates included in the corresponding primary model. Specifically, mechanical ventilation and CRRT were excluded from the 28-day models, whereas mechanical ventilation was excluded from the 1-year models because CRRT was not included in the original 1-year adjustment model. All other covariates and model specifications were retained unchanged. This analysis was considered exploratory and was used to assess the robustness of the primary estimates to the temporal definition of these organ-support covariates. Untransformed continuous, log_10_-transformed, and median-dichotomized parameterizations were also evaluated as secondary sensitivity analyses.

Subgroup analyses were conducted for the 10 standardized inflammatory-immune markers that were significantly associated with at least one mortality endpoint in the primary multivariable Cox regression analyses. Effect modification was evaluated according to age (<75 vs. ≥75 years), sex, and SA-AKI status. Age and sex were retained as the principal demographic subgroup variables, and SA-AKI was included because of its clinical and biological relevance in sepsis. Multiplicative interactions were assessed using likelihood-ratio tests comparing multivariable Cox models with and without the corresponding marker-by-subgroup interaction term. Continuous age was omitted from models evaluating interaction by age group.

RCS analyses were also treated as a main analysis of exposure-response shape. Four knots were placed at the 5th, 35th, 65th, and 95th percentiles. The selected analyses included log_10_-transformed PLR, CLR, and LHR for 28-day mortality and log_10_-transformed PLR and CLR for 1-year mortality. Curves were presented on the adjusted hazard-ratio scale with the cohort median as the reference (HR = 1). The solid curve represents the adjusted HR, the shaded area represents the 95% CI, the horizontal dashed line represents HR = 1, and the vertical dotted line identifies the cohort-median reference value.

To assess incremental prognostic performance beyond conventional severity scores, marker-by-marker nested Cox regression models were constructed. The base model included SOFA, APACHE II, SAPS II, and OASIS scores. Each candidate marker was added separately to the base model. Model fit was compared using likelihood-ratio tests. Time-dependent AUCs at 28 and 365 days were estimated using inverse probability of censoring weighting, and ΔAUC was calculated as the marker-augmented-model AUC minus the base-model AUC. The 95% confidence intervals and P values for ΔAUC were estimated using 1,000 paired bootstrap samples. IDI estimates were obtained using 1,000 perturbation-resampling iterations. For each performance measure, P values were adjusted across the 16 marker–outcome comparisons using the Benjamini–Hochberg procedure. The proportional-hazards assumption was assessed using Schoenfeld residuals. Because marker selection and performance assessment were conducted in the same cohort without optimism correction or independent validation, all performance estimates represent apparent in-sample performance.

To evaluate robustness to multiple testing, the Benjamini–Hochberg procedure was applied jointly to the 36 standardized multivariable marker–outcome tests, comprising 18 markers evaluated for two mortality endpoints. This FDR-adjusted analysis was treated as a sensitivity analysis.

To address multicollinearity and potential overadjustment, variance inflation factors (VIFs) and tolerance values were calculated separately for each marker-specific primary multivariable Cox model. Potential multicollinearity was defined as a VIF ≥5 or tolerance ≤0.20. We additionally performed a clinically specified parsimonious sensitivity analysis in which each standardized inflammatory–immune marker was entered separately and adjusted for age, sex, SOFA score, and lactate. These covariates were selected to retain basic demographic characteristics, one established measure of organ dysfunction, and a clinically relevant indicator of impaired tissue perfusion while avoiding simultaneous adjustment for multiple overlapping severity scores and physiological variables. The Benjamini–Hochberg procedure was applied jointly to the 36 marker–outcome tests from the parsimonious models. For the parsimonious sensitivity models, the marker-specific proportional-hazards assumption was evaluated by adding a marker × log(time) interaction term to each corresponding Cox model. A two-sided P<0.05 for the interaction term was considered evidence of a time-varying marker effect. For markers showing non-proportionality, extended Cox models were fitted by allowing the marker coefficient β(t) to vary as a function of log(time). These extended Cox models retained the same adjustment set as the parsimonious models: age, sex, SOFA score, and lactate. Adjusted time-varying HR(t) curves were generated. For clinical interpretability, complementary piecewise Cox models using the same covariates were fitted for days 1–90 and days 91–365.

To examine whether the mortality findings were driven mainly by deaths occurring during the index hospitalization, we performed a *post hoc* hospital-discharge landmark sensitivity analysis. For this analysis, hospital discharge served as the landmark at which eligible patients entered the risk set. Follow-up continued from hospital discharge until death or the corresponding administrative endpoint, while the underlying time scale and outcome definitions remained time since ICU admission: day 28 for the short-term analysis and day 365 for the 1-year analysis. Thus, in-hospital deaths did not enter this sensitivity analysis, and only patients discharged alive before the corresponding administrative endpoint contributed post-discharge risk time. Among the 316 patients discharged alive, 101 died after hospital discharge but before day 28 following ICU admission, and a further 49 died after discharge between days 29 and 365, resulting in 150 post-discharge deaths within 1 year. Hazard ratios are expressed per 1-SD increase in each marker. This was a *post hoc* exploratory analysis, and P values were not adjusted for multiple comparisons.

All analyses were conducted using R software (version 4.6.0; R Foundation for Statistical Computing, Vienna, Austria) and SPSS (version 26.0; IBM Corp., Armonk, NY, USA). A two-sided P value <0.05 was considered statistically significant.

## 3 Results

### 3.1 Baseline characteristics

A total of 396 patients were included in the final analysis. The median age was 72 (61, 81) years, 252 (63.6%) were male, and 167 (42.2%) met KDIGO criteria for SA-AKI. Overall, 174 patients (43.9%) died within 28 days and 230 (58.1%) died within 1 year. The median values of the key inflammatory-immune markers in the overall cohort were as follows: SII 2237.8, NLR 14.4, MLR 0.44, dNLR 9.15, NMLR 15.0, SIRI 4.9, PIV 695.0, PLR 220.2, CLR 217.6, HALP 12.0, and LHR 1.0. One-year vital status was ascertained for all 396 included patients.

Compared with 28-day survivors, non-survivors were older, had lower DBP and MAP, lower 24-h urine output, lower lymphocyte count, lower platelet count, lower PCT, higher LDL-C, higher BNP, higher troponin T, higher illness severity scores, lower GCS, and higher rates of mechanical ventilation and CRRT (all P<0.05). SA-AKI did not differ significantly between 28-day non-survivors and survivors (47.7% vs. 37.8%, P = 0.061). Among the inflammatory-immune indices, nonsurvivors had higher NLR, dNLR, NMLR, and PLR, but lower HALP and LHR (**Table 1**; **Supplementary Table S1**).

**Table 1:** Clinical characteristics stratified by all-cause mortality.

Variables	Total population	All-cause mortality_28-day_			All-cause mortality_1-year_		
		No	Yes	P value	No	Yes	P value
N	396	222	174		166	230	
Age, years	72.00 (61.00, 81.00)	69.00 (58.00, 77.00)	76.00 (66.00, 83.75)	<0.001	67.00 (56.00, 74.00)	76.00 (67.00, 85.00)	<0.001
Sex, n (%)				0.962			0.277
Male	252 (63.6)	142 (64.0)	110 (63.2)		100 (60.2)	152 (66.1)	
Female	144 (36.4)	80 (36.0)	64 (36.8)		66 (39.8)	78 (33.9)	
SA-AKI, n (%)				0.061			0.079
Yes	167 (42.2)	84 (37.8)	83 (47.7)		61 (36.7)	106 (46.1)	
No	229 (57.8)	138 (62.2)	91 (52.3)		105 (63.3)	124 (53.9)	
CHF, n (%)				0.066			0.017
Yes	51 (12.9)	22 (9.9)	29 (16.7)		13 (7.8)	38 (16.5)	
No	345 (87.1)	200 (90.1)	145 (83.3)		153 (92.2)	192 (83.5)	
Cerebrovascular disease, n (%)				0.735			0.021
Yes	159 (40.2)	87 (39.2)	72 (41.4)		55 (33.1)	104 (45.2)	
No	237 (59.8)	135 (60.8)	102 (58.6)		111 (66.9)	126 (54.8)	
Diabetes, n (%)				0.573			0.484
Yes	116 (29.3)	62 (27.9)	54 (31.0)		45 (27.1)	71 (30.9)	
No	280 (70.7)	160 (72.1)	120 (69.0)		121 (72.9)	159 (69.1)	
ARDS, n (%)				1.000			1.000
Yes	35 (8.8)	20 (9.0)	15 (8.6)		15 (9.0)	20 (8.7)	
No	361 (91.2)	202 (91.0)	159 (91.4)		151 (91.0)	210 (91.3)	
SOFA_24h_	8.00 (5.00, 11.00)	7.00 (5.00, 10.00)	9.00 (6.00, 13.00)	<0.001	7.00 (5.00, 9.00)	9.00 (6.00, 12.00)	<0.001
APACHE II_24h_	22.00 (17.00, 27.00)	19.00 (14.00, 24.00)	24.00 (20.00, 30.00)	<0.001	18.00 (13.00, 23.00)	24.00 (19.00, 29.00)	<0.001
GCS_24h_	12.00 (6.00, 15.00)	13.00 (7.00, 15.00)	10.00 (4.00, 15.00)	<0.001	15.00 (8.00, 15.00)	10.00 (4.00, 15.00)	<0.001
SAPS II score_24h_	50.00 (38.00, 63.00)	45.00 (34.00, 58.00)	58.00 (47.00, 68.50)	<0.001	43.00 (32.00, 54.00)	57.00 (47.00, 67.00)	<0.001
SIRS_24h_	3.00 (2.00, 3.00)	3.00 (2.00, 3.00)	3.00 (2.00, 3.00)	0.064	3.00 (2.00, 3.00)	3.00 (2.00, 3.00)	0.303
OASIS_24h_	39.00 (32.00, 45.00)	37.00 (30.00, 44.00)	42.00 (35.00, 46.00)	<0.001	36.00 (30.00, 44.00)	41.00 (35.00, 46.00)	<0.001
Mechanical ventilation_48h_, n (%)				<0.001			<0.001
Yes	350 (88.4)	184 (82.9)	166 (95.4)		134 (80.7)	216 (93.9)	
No	46 (11.6)	38 (17.1)	8 (4.6)		32 (19.3)	14 (6.1)	
CRRT_48h_, n (%)				0.020			0.448
Yes	70 (17.7)	30 (13.5)	40 (23.0)		26 (15.7)	44 (19.1)	
No	326 (82.3)	192 (86.5)	134 (77.0)		140 (84.3)	186 (80.9)	
Standardized SII	-0.2957 (-0.4716, 0.0780)	-0.3227 (-0.4738, -0.0213)	-0.2477 (-0.4719, 0.1882)	0.213	-0.2943 (-0.4575, -0.0088)	-0.3045 (-0.4822, 0.1567)	0.803
Standardized NLR	-0.3331 (-0.5971, 0.2840)	-0.4077 (-0.6198, 0.2272)	-0.2133 (-0.5300, 0.4082)	0.014	-0.3628 (-0.5985, 0.2389)	-0.2988 (-0.5980, 0.3280)	0.508
Standardized dNLR	−0.2784 (−0.7350–0.4681)	−0.3725 (−0.7676–0.4494)	−0.1863 (−0.6184–0.7073)	0.032	−0.2725 (−0.7274–0.5021)	−0.2784 (−0.7453–0.4461)	0.907
Standardized MLR	−0.3265 (−0.6040–0.2336)	−0.3833 (−0.6172–0.1968)	−0.2348 (−0.5660–0.3413)	0.065	−0.3946 (−0.6424–0.1626)	−0.2640 (−0.5660–0.2978)	0.069
Standardized NMLR	-0.3301 (-0.5996, 0.2831)	-0.3943 (-0.6206, 0.2205)	-0.2067 (-0.5289, 0.3980)	0.015	-0.3745 (-0.6047, 0.2390)	-0.3008 (-0.5977, 0.3171)	0.480
Standardized SIRI	-0.2644 (-0.4155, 0.0420)	-0.3108 (-0.4223, 0.0218)	-0.2202 (-0.4051, 0.0684)	0.142	-0.2945 (-0.4236, 0.0291)	-0.2517 (-0.4096, 0.0493)	0.425
Standardized PIV	-0.2384 (-0.3182, -0.0139)	-0.2427 (-0.3131, -0.0367)	-0.2347 (-0.3238, 0.0213)	0.663	-0.2476 (-0.3152, -0.0367)	-0.2345 (-0.3195, 0.0005)	0.667
Standardized PLR	-0.3712 (-0.5717, 0.2834)	-0.4138 (-0.5841, 0.0567)	-0.2728 (-0.5461, 0.4714)	0.028	-0.4138 (-0.5642, 0.0363)	-0.3087 (-0.6031, 0.3916)	0.174
Standardized NPR	-0.2367 (-0.3268, -0.0535)	-0.2548 (-0.3425, -0.0773)	-0.2039 (-0.3093, -0.0483)	0.058	-0.2458 (-0.3420, -0.0212)	-0.2323 (-0.3175, -0.0764)	0.769
Standardized PAR	-0.1493 (-0.7133, 0.4408)	-0.0852 (-0.6794, 0.5927)	-0.2171 (-0.7333, 0.3731)	0.128	-0.1013 (-0.7298, 0.5251)	-0.1935 (-0.7108, 0.4190)	0.768
Standardized CAR	-0.1079 (-0.8563, 0.6460)	-0.0354 (-0.7313, 0.6678)	-0.2401 (-0.9457, 0.5888)	0.187	0.0727 (-0.6591, 0.7585)	-0.2061 (-0.9457, 0.4732)	0.013
Standardized CLR	-0.3176 (-0.5875, 0.1518)	-0.3343 (-0.5835, 0.0914)	-0.2925 (-0.5964, 0.3421)	0.338	-0.3049 (-0.5546, 0.1534)	-0.3420 (-0.6019, 0.1434)	0.427
Standardized CALLY	-0.1687 (-0.1908, -0.0967)	-0.1672 (-0.1891, -0.0970)	-0.1715 (-0.1935, -0.0947)	0.252	-0.1698 (-0.1904, -0.1125)	-0.1667 (-0.1908, -0.0753)	0.556
Standardized HALP	-0.2162 (-0.3429, 0.0039)	-0.1538 (-0.3236, 0.0222)	-0.2512 (-0.3650, -0.0316)	0.011	-0.1538 (-0.3169, 0.0166)	-0.2460 (-0.3596, -0.0063)	0.038
Standardized NHR	-0.2796 (-0.5959, 0.2216)	-0.3188 (-0.6012, 0.1059)	-0.2386 (-0.5837, 0.2791)	0.562	-0.3070 (-0.5970, 0.2523)	-0.2767 (-0.5965, 0.1796)	0.592
Standardized MHR	-0.3356 (-0.5980, 0.2240)	-0.2869 (-0.5652, 0.2150)	-0.3929 (-0.6532, 0.2465)	0.199	-0.3474 (-0.5712, 0.2150)	-0.2974 (-0.6300, 0.2431)	0.780
Standardized LHR	-0.3010 (-0.6143, 0.2243)	-0.1814 (-0.5684, 0.2830)	-0.3767 (-0.7291, 0.1509)	0.006	-0.2364 (-0.5613, 0.1614)	-0.3521 (-0.6833, 0.2870)	0.190
Standardized PHR	-0.2612 (-0.6101, 0.3207)	-0.1771 (-0.5929, 0.3592)	-0.3140 (-0.6165, 0.1881)	0.087	-0.2340 (-0.6253, 0.3592)	-0.2798 (-0.5974, 0.2901)	0.454

SA-AKI, Sepsis-associated acute kidney injury; CHF, Chronic heart failure; ARDS, Acute respiratory distress syndrome; SOFA, Sequential organ failure assessment; APACHE II, Acute physiology and chronic health evaluation II; GCS, Glasgow coma scale; SAPS II, Simplified acute physiology score II_;_ SIRS, Systemic inflammatory response syndrome_;_ OASIS, Oxford acute severity of illness score; CRRT, continuous renal replacement therapy; SII, Systemic immune-inflammation index; NLR, Neutrophil-to-lymphocyte ratio; dNLR, Derived neutrophil-to-lymphocyte ratio; MLR, Monocyte-to-lymphocyte ratio; NMLR, Neutrophil plus monocyte-to-lymphocyte ratio; SIRI, Systemic inflammation response index; PIV, Pan-immune-inflammation value; PLR, Platelet-to-lymphocyte ratio; NPR, Neutrophil-to-platelet ratio; PAR, Platelet-to-albumin ratio; CAR, C-reactive protein-to-albumin ratio; CLR, C-reactive protein-to-lymphocyte ratio; CALLY, C-reactive protein-albumin-lymphocyte index; HALP, Hemoglobin-albumin-lymphocyte-platelet score; NHR, Neutrophil-to-high-density lipoprotein cholesterol ratio; MHR, Monocyte-to-high-density lipoprotein cholesterol ratio; LHR, Lymphocyte-to-high-density lipoprotein cholesterol ratio; PHR, Platelet-to-high-density lipoprotein cholesterol ratio.

Compared with 1-year survivors, nonsurvivors were older, more frequently had CHF and cerebrovascular disease, and had lower DBP and 24-h urine output. They had higher serum potassium, LDL-C, BNP, and troponin T, but unexpectedly lower CRP and PCT. One-year nonsurvivors also had higher severity scores, lower GCS scores, and more frequent mechanical ventilation, as well as lower CAR and HALP (all P<0.05) (**Table 1**; **Supplementary Table S1**).

### 3.2 Univariate Cox regression

For 28-day all-cause mortality, significant clinical covariates included age, DBP, HR, MAP, ICU urine output_24h_, SaO_2_, PaCO_2_, pH, lactate, serum potassium, lymphocyte count, platelet count, PCT, BNP, ALT, albumin, prothrombin time, D-dimer, SOFA, APACHE II, GCS, SAPS II, SIRS, OASIS, mechanical ventilation, and CRRT (all P<0.05).

For the inflammatory-immune indices, each 1-SD increase in SII (HR 1.166; P = 0.003), NLR (HR 1.177; P = 0.002), MLR (HR 1.205; P = 0.006), NMLR (HR 1.179; P = 0.002), SIRI (HR 1.192; P = 0.007), PIV (HR 1.206; P = 0.002), PLR (HR 1.253; P<0.001), and CLR (HR 1.217; P = 0.002) was associated with higher mortality (**Supplementary Table S3**).

For 1-year all-cause mortality, significant clinical covariates included age, CHF, DBP, 24-h urine output, SaO_2_, PCO_2_, lactate, serum potassium, CRP, PCT, LDL-C, BNP, ALT, SOFA, APACHE II, GCS, SAPS II, OASIS, and mechanical ventilation (all P<0.05). For the inflammatory-immune indices, each 1-SD increase in SII (HR 1.147; P = 0.010), NLR (HR 1.138; P = 0.023), MLR (HR 1.165; P = 0.018), NMLR (HR 1.140; P = 0.021), PIV (HR 1.170; P = 0.014), and PLR (HR 1.233; P<0.001) was associated with higher mortality (**Supplementary Table S3**).

### 3.3 Multivariate Cox regression

After multivariable adjustment, each 1-SD increase in SII (HR 1.157; P = 0.004), NLR (HR 1.141; P = 0.012), dNLR (HR 1.171; P = 0.017), MLR (HR 1.141; P = 0.039), NMLR (HR 1.142; P = 0.012), PIV (HR 1.214; P = 0.002), PLR (HR 1.253; P<0.001), and CLR (HR 1.313; P<0.001) was associated with higher 28-day mortality. Conversely, standardized LHR (HR 0.759; P = 0.005) and PHR (HR 0.814; P = 0.028) were inversely associated (**Table 2**). In median-dichotomized analyses, high SII, NLR, dNLR, MLR, NMLR, SIRI, and PLR were associated with higher mortality, whereas high MHR, HALP, LHR, and PHR were associated with lower mortality (all P<0.05). Significant log_10_-transformed associations were also observed for NLR, dNLR, MLR, NMLR, PLR, HALP, LHR, and PHR (**Supplementary Table S4**).

**Table 2:** Multivariate Cox regression analysis for all-cause mortality.

Variables	All-cause mortality_28-day_			All-cause mortality_1-year_		
	HR	95% CI	P value	HR	95% CI	P value
Standardized SII	1.157	1.048-1.277	0.004	1.109	1.005-1.224	0.039
Standardized NLR	1.141	1.029-1.265	0.012	1.091	0.978-1.217	0.118
Standardized dNLR	1.171	1.028–1.334	0.017	1.137	1.006–1.284	0.039
Standardized MLR	1.141	1.007–1.293	0.039	1.079	0.947–1.228	0.254
Standardized NMLR	1.142	1.030-1.267	0.012	1.092	0.978-1.218	0.118
Standardized SIRI	1.108	0.948-1.294	0.196	1.082	0.919-1.275	0.343
Standardized PIV	1.214	1.075-1.371	0.002	1.163	1.014-1.335	0.031
Standardized PLR	1.253	1.114-1.410	<0.001	1.193	1.071-1.329	0.001
Standardized NPR	1.045	0.905-1.206	0.548	1.043	0.908-1.198	0.554
Standardized PAR	0.888	0.742-1.063	0.196	0.925	0.794-1.078	0.320
Standardized CAR	0.992	0.835-1.178	0.924	0.974	0.840-1.130	0.728
Standardized CLR	1.313	1.145-1.505	<0.001	1.258	1.104-1.435	<0.001
Standardized CALLY	0.990	0.808-1.212	0.921	1.005	0.874-1.155	0.947
Standardized HALP	1.046	0.922-1.186	0.483	1.069	0.955-1.198	0.247
Standardized NHR	0.870	0.748-1.012	0.072	0.946	0.833-1.073	0.385
Standardized MHR	0.905	0.775-1.058	0.211	0.945	0.821-1.087	0.426
Standardized LHR	0.759	0.626-0.920	0.005	0.819	0.703-0.954	0.010
Standardized PHR	0.814	0.677-0.978	0.028	0.902	0.777-1.047	0.177

For all-cause mortality_28-day_, multivariate Cox regression analysis adjusted for age, heart rate, mean arterial pressure, ICU urine output_24h_, arterial oxygen saturation, PaCO_2_, Ph, lactate, serum potassium, PCT, BNP, ALT, Prothrombin time, D-dimer, SOFA_24h_, APACHE II_24h_, GCS_24h_, SAPS II score_24h_, SIRS_24h_, OASIS_24h_, Mechanical ventilation_48h_, CRRT_48h_.

For all-cause mortality_1-year_, multivariate Cox regression analysis adjusted for age, ICU urine output_24h_, PaCO_2_, arterial oxygen saturation, CRP, PCT, lactate, serum potassium, ALT, BNP, SOFA_24h_, APACHE II_24h_, GCS_24h_, SAPS II score_24h_, OASIS_24h_, and mechanical ventilation_48h_. Because CRP is a component of CAR, CLR, and CALLY, the multivariable models for these three indices excluded CRP as a covariate to avoid mathematical coupling.SII, Systemic immune-inflammation index; NLR, Neutrophil-to-lymphocyte ratio; dNLR, Derived neutrophil-to-lymphocyte ratio; MLR, Monocyte-to-lymphocyte ratio; NMLR, Neutrophil plus monocyte-to-lymphocyte ratio; SIRI, Systemic inflammation response index; PIV, Pan-immune-inflammation value; PLR, Platelet-to-lymphocyte ratio; NPR, Neutrophil-to-platelet ratio; PAR, Platelet-to-albumin ratio; CAR, C-reactive protein-to-albumin ratio; CLR, C-reactive protein-to-lymphocyte ratio; CALLY, C-reactive protein-albumin-lymphocyte index; HALP, Hemoglobin-albumin-lymphocyte-platelet score; NHR, Neutrophil-to-high-density lipoprotein cholesterol ratio; MHR, Monocyte-to-high-density lipoprotein cholesterol ratio; LHR, Lymphocyte-to-high-density lipoprotein cholesterol ratio; PHR, Platelet-to-high-density lipoprotein cholesterol ratio.

For 1-year mortality, each 1-SD increase in SII (HR 1.109; P = 0.039), dNLR (HR 1.137; P = 0.039), PIV (HR 1.163; P = 0.031), PLR (HR 1.193; P = 0.001), and CLR (HR 1.258; P<0.001) was associated with higher mortality, whereas standardized LHR was inversely associated (HR 0.819; P = 0.010) (**Table 2**). High versus low NLR (HR 1.443; P = 0.007), NMLR (HR 1.437; P = 0.007), PLR (HR 1.470; P = 0.005) were positively associated with mortality, whereas high HALP (HR 0.694; P = 0.007) and LHR (HR 0.682; P = 0.005) were inversely associated. Among the log_10_-transformed markers, only log_10_ NMLR was additionally associated with 1-year mortality (HR 1.336; P = 0.047) (**Supplementary Table S4**).

Alternative parameterizations produced a mixed pattern of robustness. PLR remained associated with higher mortality after median dichotomization at both 28 days (HR 1.464, 95% CI 1.074-1.994; P = 0.016) and 1 year (HR 1.470, 95% CI 1.125-1.919; P = 0.005), while high LHR remained inversely associated at 28 days (HR 0.628, 95% CI 0.462-0.854; P = 0.003) and 1 year (HR 0.682, 95% CI 0.523-0.890; P = 0.005). In contrast, although standardized PIV was associated with both endpoints in the primary models, neither its log10-transformed nor median-dichotomized form was significant for either endpoint (all P≥0.306), demonstrating parameterization dependence (**Supplementary Table S4**).

### 3.4 Subgroup and interaction analyses

Exploratory subgroup analyses evaluated effect modification by age, sex, and SA-AKI status for the 10 standardized inflammatory-immune markers that were significantly associated with at least one mortality endpoint in the primary multivariable Cox regression analyses. For 28-day mortality, CLR showed directionally similar positive associations in both patients aged <75 years (HR 1.306, 95% CI 1.110-1.537) and those aged ≥75 years (HR 1.254, 95% CI 0.934-1.685; P for interaction=0.499). PLR was likewise positively associated in both age strata (HR 1.141, 95% CI 0.955-1.364 for <75 years; HR 1.246, 95% CI 1.015-1.531 for ≥75 years; P for interaction=0.924), whereas LHR was inversely associated in both strata (HR 0.851, 95% CI 0.642-1.128 for <75 years; HR 0.690, 95% CI 0.509-0.933 for ≥75 years; P for interaction=0.424). No formal interaction was detected for CLR, PLR, or LHR by age, sex, or SA-AKI status at either endpoint (all P for interaction >0.05). Three PIV interactions reached nominal statistical significance: age for 28-day mortality (P for interaction=0.029), age for 1-year mortality (P for interaction=0.003), and SA-AKI status for 1-year mortality (P for interaction=0.037). These findings were exploratory and were not adjusted for multiple testing, and the directionally divergent PIV estimates should not be interpreted as confirmed effect modification without external validation. Complete stratum-specific hazard ratios and interaction P values are presented in **Table 3**.

**Table 3:** Subgroup analysis of standardized inflammatory-immune markers by age, sex, and SA-AKI status.

Outcome	Marker	Stratifying variable	Subgroup	HR	95% CI	P value	P for interaction
All-cause mortality_28-day_	standardized SII	Age	<75 years	1.144	1.024-1.278	0.017	0.483
			≥75 years	1.105	0.840-1.452	0.475	
		Sex	Male	1.170	1.056-1.297	0.003	0.895
			Female	1.158	0.841-1.596	0.368	
		SA-AKI	Yes	1.016	0.742-1.390	0.923	0.593
			No	1.089	0.946-1.255	0.236	
	standardized NLR	Age	<75 years	1.048	0.884-1.243	0.587	0.424
			≥75 years	1.103	0.884-1.376	0.385	
		Sex	Male	1.162	1.045-1.293	0.006	0.635
			Female	1.115	0.763-1.631	0.573	
		SA-AKI	Yes	1.002	0.783-1.283	0.987	0.311
			No	1.090	0.939-1.265	0.256	
	standardized dNLR	Age	<75 years	1.040	0.821-1.317	0.744	0.645
			≥75 years	1.164	0.918-1.475	0.209	
		Sex	Male	1.244	1.075-1.440	0.003	0.673
			Female	1.176	0.848-1.631	0.332	
		SA-AKI	Yes	1.044	0.824-1.322	0.723	0.138
			No	1.184	0.938-1.494	0.156	
	standardized MLR	Age	<75 years	1.151	0.950-1.395	0.152	0.147
			≥75 years	1.000	0.795-1.259	0.997	
		Sex	Male	1.228	1.054-1.432	0.009	0.222
			Female	1.078	0.837-1.389	0.562	
		SA-AKI	Yes	0.918	0.696-1.210	0.543	0.185
			No	1.177	0.985-1.406	0.073	
	standardized NMLR	Age	<75 years	1.051	0.886-1.247	0.570	0.408
			≥75 years	1.100	0.883-1.372	0.395	
		Sex	Male	1.164	1.046-1.296	0.005	0.626
			Female	1.115	0.766-1.624	0.569	
		SA-AKI	Yes	1.000	0.781-1.281	0.999	0.302
			No	1.092	0.941-1.268	0.247	
	standardized PIV	Age	<75 years	1.240	1.114-1.382	<0.001	0.029
			≥75 years	0.794	0.496-1.268	0.334	
		Sex	Male	1.274	1.128-1.438	<0.001	0.157
			Female	0.873	0.536-1.420	0.583	
		SA-AKI	Yes	0.762	0.464-1.252	0.283	0.069
			No	1.211	1.050-1.396	0.008	
	standardized PLR	Age	<75 years	1.141	0.955-1.364	0.147	0.924
			≥75 years	1.246	1.015-1.531	0.036	
		Sex	Male	1.376	1.184-1.599	<0.001	0.548
			Female	1.220	0.957-1.556	0.108	
		SA-AKI	Yes	1.265	0.988-1.619	0.062	0.900
			No	1.164	1.009-1.343	0.037	
	standardized CLR	Age	<75 years	1.306	1.110-1.537	0.001	0.499
			≥75 years	1.254	0.934-1.685	0.132	
		Sex	Male	1.264	1.027-1.556	0.027	0.672
			Female	1.240	1.000-1.538	0.050	
		SA-AKI	Yes	1.322	1.115-1.567	0.001	0.213
			No	1.269	1.004-1.603	0.046	
	standardized LHR	Age	<75 years	0.851	0.642-1.128	0.261	0.424
			≥75 years	0.690	0.509-0.933	0.016	
		Sex	Male	0.678	0.523-0.878	0.003	0.562
			Female	0.765	0.567-1.033	0.080	
		SA-AKI	Yes	0.808	0.623-1.047	0.107	0.745
			No	0.737	0.549-0.989	0.042	
	standardized PHR	Age	<75 years	0.887	0.697-1.128	0.327	0.355
			≥75 years	0.708	0.513-0.978	0.036	
		Sex	Male	0.787	0.640-0.969	0.024	0.962
			Female	0.835	0.557-1.252	0.383	
		SA-AKI	Yes	0.812	0.626-1.054	0.118	0.847
			No	0.889	0.662-1.193	0.433	
All-cause mortality_1-year_	standardized SII	Age	<75 years	1.110	0.985-1.249	0.086	0.256
			≥75 years	0.987	0.763-1.276	0.921	
		Sex	Male	1.126	1.016-1.249	0.024	0.547
			Female	1.010	0.752-1.355	0.949	
		SA-AKI	Yes	1.070	0.846-1.354	0.570	0.853
			No	1.103	0.977-1.247	0.114	
	standardized NLR	Age	<75 years	1.081	0.932-1.252	0.303	0.404
			≥75 years	1.064	0.866-1.307	0.555	
		Sex	Male	1.119	1.008-1.242	0.035	0.485
			Female	1.043	0.763-1.426	0.792	
		SA-AKI	Yes	1.084	0.872-1.346	0.467	0.473
			No	1.111	0.975-1.265	0.113	
	standardized dNLR	Age	<75 years	1.137	0.927-1.395	0.219	0.355
			≥75 years	1.116	0.909-1.370	0.293	
		Sex	Male	1.190	1.040-1.363	0.011	0.357
			Female	1.097	0.833-1.444	0.510	
		SA-AKI	Yes	1.159	0.952-1.410	0.142	0.383
			No	1.182	0.972-1.438	0.093	
	standardized MLR	Age	<75 years	1.121	0.941-1.336	0.201	0.119
			≥75 years	0.944	0.759-1.173	0.602	
		Sex	Male	1.151	0.960-1.380	0.130	0.569
			Female	1.074	0.870-1.324	0.507	
		SA-AKI	Yes	0.918	0.710-1.188	0.516	0.146
			No	1.162	0.987-1.369	0.072	
	standardized NMLR	Age	<75 years	1.083	0.933-1.256	0.294	0.386
			≥75 years	1.061	0.864-1.303	0.571	
		Sex	Male	1.120	1.008-1.244	0.035	0.487
			Female	1.045	0.767-1.424	0.779	
		SA-AKI	Yes	1.081	0.870-1.343	0.484	0.457
			No	1.113	0.977-1.269	0.109	
	standardized PIV	Age	<75 years	1.223	1.087-1.376	<0.001	0.003
			≥75 years	0.652	0.427-0.997	0.048	
		Sex	Male	1.237	1.090-1.404	<0.001	0.084
			Female	0.865	0.586-1.275	0.463	
		SA-AKI	Yes	0.827	0.568-1.203	0.320	0.037
			No	1.250	1.097-1.424	<0.001	
	standardized PLR	Age	<75 years	1.153	1.007-1.322	0.040	0.751
			≥75 years	1.222	0.989-1.511	0.064	
		Sex	Male	1.354	1.180-1.552	<0.001	0.186
			Female	1.064	0.852-1.327	0.586	
		SA-AKI	Yes	1.209	0.975-1.499	0.083	0.741
			No	1.161	1.006-1.341	0.042	
	standardized CLR	Age	<75 years	1.264	1.082-1.476	0.003	0.538
			≥75 years	1.218	0.930-1.596	0.152	
		Sex	Male	1.289	1.078-1.542	0.005	0.577
			Female	1.253	1.029-1.527	0.025	
		SA-AKI	Yes	1.202	1.008-1.433	0.041	0.076
			No	1.306	1.053-1.620	0.015	
	standardized LHR	Age	<75 years	0.915	0.731-1.146	0.440	0.387
			≥75 years	0.783	0.621-0.986	0.038	
		Sex	Male	0.787	0.633-0.979	0.032	0.590
			Female	0.839	0.660-1.066	0.151	
		SA-AKI	Yes	0.895	0.727-1.101	0.294	0.383
			No	0.799	0.628-1.018	0.069	
	standardized PHR	Age	<75 years	0.968	0.805-1.164	0.731	0.427
			≥75 years	0.812	0.630-1.046	0.107	
		Sex	Male	0.930	0.796-1.086	0.359	0.249
			Female	0.725	0.499-1.053	0.091	
		SA-AKI	Yes	0.965	0.807-1.154	0.695	0.161
			No	0.857	0.659-1.115	0.251	

For all-cause mortality_28-day_, multivariate Cox regression analysis adjusted for age, heart rate, mean arterial pressure, ICU urine output_24h_, arterial oxygen saturation, PaCO_2_, Ph, lactate, serum potassium, PCT, BNP, ALT, Prothrombin time, D-dimer, SOFA_24h_, APACHE II_24h_, GCS_24h_, SAPS II score_24h_, SIRS_24h_, OASIS_24h_, Mechanical ventilation_48h_, CRRT_48h_.

For all-cause mortality_1-year_, multivariate Cox regression analysis adjusted for age, ICU urine output_24h_, PaCO_2_, arterial oxygen saturation, CRP, PCT, lactate, serum potassium, ALT, BNP, SOFA_24h_, APACHE II_24h_, GCS_24h_, SAPS II score_24h_, OASIS_24h_, and mechanical ventilation_48h_. Because CRP is a component of CLR, the multivariable models for CLR excluded CRP as a covariate to avoid mathematical coupling.

SII, Systemic immune-inflammation index; NLR, Neutrophil-to-lymphocyte ratio; dNLR, Derived neutrophil-to-lymphocyte ratio; MLR, Monocyte-to-lymphocyte ratio; NMLR, Neutrophil plus monocyte-to-lymphocyte ratio; SIRI, Systemic inflammation response index; PIV, Pan-immune-inflammation value; PLR, Platelet-to-lymphocyte ratio; NPR, Neutrophil-to-platelet ratio; PAR, Platelet-to-albumin ratio; CAR, C-reactive protein-to-albumin ratio; CLR, C-reactive protein-to-lymphocyte ratio; CALLY, C-reactive protein-albumin-lymphocyte index; HALP, Hemoglobin-albumin-lymphocyte-platelet score; NHR, Neutrophil-to-high-density lipoprotein cholesterol ratio; MHR, Monocyte-to-high-density lipoprotein cholesterol ratio; LHR, Lymphocyte-to-high-density lipoprotein cholesterol ratio; PHR, Platelet-to-high-density lipoprotein cholesterol ratio.

### 3.5 Restricted cubic spline analysis

RCS analyses showed statistically significant nonlinear associations of log_10_-transformed PLR and CLR with 28-day mortality. For PLR, P-overall=0.003 and P-nonlinear=0.005; for CLR, P-overall=0.001 and P-nonlinear=0.002. Log_10_-transformed LHR was inversely associated with 28-day mortality (P-overall<0.001), with no evidence of nonlinearity (P-nonlinear=0.811), supporting an approximately linear inverse association (**Figure 2**).

**Figure 2:**
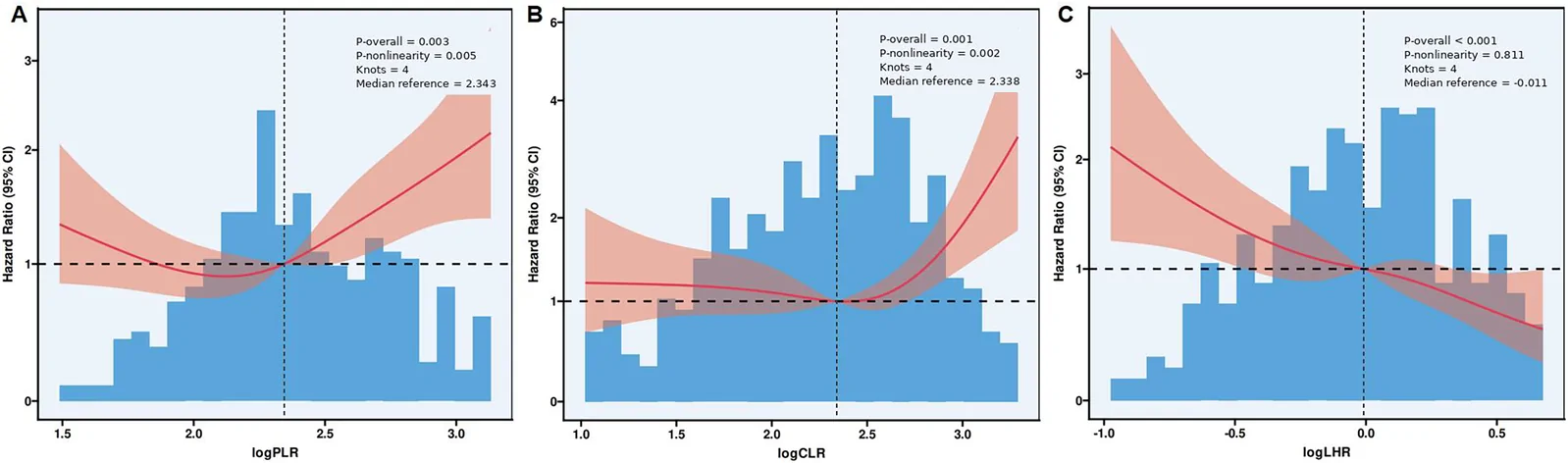
RCS plots showing the correlation between log_10_-transformed PLR **(A)**, CLR **(B)**, LHR **(C)** and all-cause mortality28-day. The solid red curve represents the adjusted hazard ratio and the shaded band its 95% CI. The horizontal dashed line indicates HR = 1, the vertical dotted line indicates the cohort-median reference value, and the blue histogram shows the marker distribution. Four knots were placed at the 5th, 35th, 65th, and 95th percentiles. RCS, restricted cubic spline; CI, confidence interval; PLR, platelet-to-lymphocyte ratio; CLR, C-reactive protein-to-lymphocyte ratio; LHR, lymphocyte-to-high-density lipoprotein cholesterol ratio.

For 1-year mortality, both log_10_-transformed PLR (P-overall=0.007; P-nonlinear=0.006) and CLR (P-overall=0.001; P-nonlinear=0.004) showed significant nonlinear associations (**Figure 3**). The y-axis represents the adjusted hazard ratio; vertical dotted lines indicate cohort-median reference values; knot positions (5th, 35th, 65th, 95th percentiles) and P-overall/P-nonlinear are annotated in each panel.

**Figure 3:**
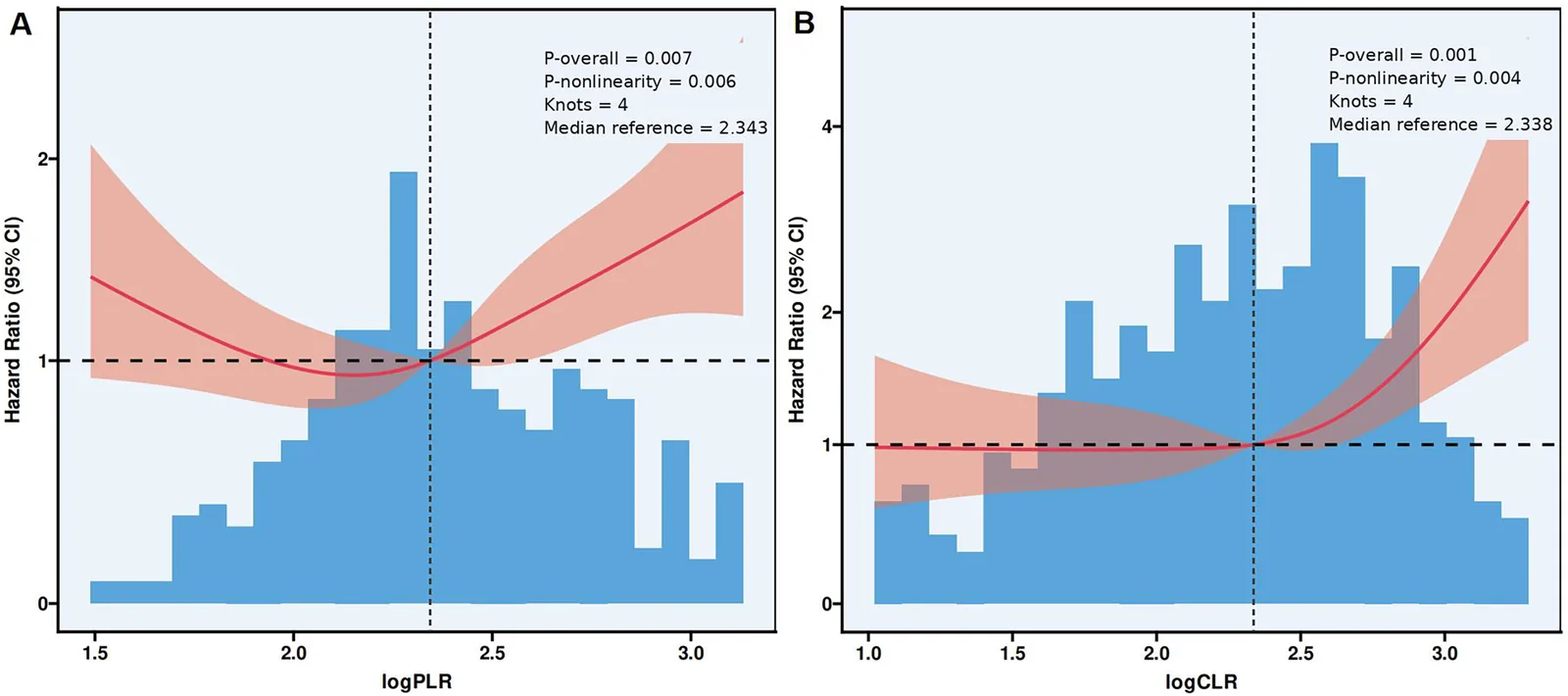
RCS plots showing the correlation between log_10_-transformed PLR **(A)**, CLR **(B)** and all-cause mortality1-year. The solid red curve represents the adjusted hazard ratio and the shaded band its 95% CI. The horizontal dashed line indicates HR = 1, the vertical dotted line indicates the cohort-median reference value, and the blue histogram shows the marker distribution. Four knots were placed at the 5th, 35th, 65th, and 95th percentiles. RCS, restricted cubic spline; CI, confidence interval; PLR, platelet-to-lymphocyte ratio; CLR, C-reactive protein-to-lymphocyte ratio.

### 3.6 Incremental prognostic value beyond severity scores

All nested-model comparisons included 396 patients, with 174 deaths within 28 days and 230 deaths within 1 year. The time-dependent AUCs of the severity-score base models were 0.719 (95% CI 0.669–0.769) for 28-day mortality and 0.745 (95% CI 0.697–0.794) for 1-year mortality.

For 28-day mortality, adding SII (q=0.041), PIV (q=0.034), PLR (q=0.012), or LHR (q=0.041) significantly improved overall model fit according to the FDR-adjusted likelihood ratio tests. For 1-year mortality, corresponding improvements were observed after adding SII (q=0.047), PIV (q=0.0497), or PLR (q=0.012). However, none of the marker-specific improvements in time-dependent AUC or IDI remained statistically significant after FDR correction. PLR produced the largest apparent increase in AUC for both 28-day mortality (ΔAUC 0.019, 95% CI 0.003–0.041; P = 0.012, q=0.112) and 1-year mortality (ΔAUC 0.013, 95% CI 0.001–0.029; P = 0.022, q=0.117), but neither improvement remained significant after correction. The complete results are presented in **Table 4**.

**Table 4:** Marker-by-marker incremental prognostic performance of inflammatory–immune markers beyond conventional severity scores.

Outcome	Marker-augmented model	AUC (95% CI)	ΔAUC (95% CI); P/q	LR chi-square (df); P/q	IDI (95% CI); P/q
28-day mortality	SOFA + APACHE II + SAPS II + OASIS	0.719 (0.669-0.769)	—	—	—
	Base model + standardized SII	0.731 (0.682-0.780)	0.012 (0.001-0.034) P/q = 0.014/0.112	6.191 (1) P/q = 0.013/0.041	0.0135 (-0.0050-0.0328) P/q = 0.104/0.220
	Base model + standardized NLR	0.727 (0.678-0.777)	0.009 (0.000-0.026) P/q = 0.036/0.121	3.686 (1) P/q = 0.055/0.090	0.0096 (-0.0006-0.0240) P/q = 0.068/0.220
	Base model + standardized dNLR	0.726 (0.677-0.776)	0.008 (-0.001-0.024) P/q = 0.152/0.347	3.652 (1) P/q = 0.056/0.090	0.0106 (-0.0008-0.0345) P/q = 0.086/0.220
	Base model + standardized MLR	0.724 (0.674-0.774)	0.005 (-0.002-0.020) P/q = 0.250/0.428	2.539 (1) P/q = 0.111/0.127	0.0053 (-0.0054-0.0229) P/q = 0.264/0.325
	Base model + standardized NMLR	0.727 (0.678-0.777)	0.009 (0.000-0.026) P/q = 0.038/0.121	3.724 (1) P/q = 0.054/0.090	0.0097 (-0.0009-0.0258) P/q = 0.076/0.220
	Base model + standardized PIV	0.724 (0.674-0.774)	0.005 (-0.003-0.019) P/q = 0.268/0.428	7.458 (1) P/q = 0.006/0.034	0.0089 (-0.0019-0.0237) P/q = 0.118/0.220
	Base model + standardized PLR	0.738 (0.689-0.787)	0.019 (0.003-0.041) P/q = 0.012/0.112	10.158 (1) P/q = 0.001/0.012	0.0217 (-0.0031-0.0475) P/q = 0.068/0.220
	Base model + standardized CLR	0.722 (0.672-0.772)	0.003 (-0.004-0.016) P/q = 0.460/0.613	2.560 (1) P/q = 0.110/0.127	0.0039 (-0.0032-0.0173) P/q = 0.252/0.325
	Base model + standardized LHR	0.728 (0.679-0.778)	0.010 (-0.002-0.030) P/q = 0.142/0.347	6.265 (1) P/q = 0.012/0.041	0.0128 (-0.0011-0.0430) P/q = 0.084/0.220
	Base model + standardized PHR	0.717 (0.667-0.768)	-0.002 (-0.007-0.015) P/q = 0.995/0.995	2.717 (1) P/q = 0.099/0.127	0.0048 (-0.0011-0.0232) P/q = 0.212/0.325
1-year mortality	SOFA + APACHE II + SAPS II + OASIS	0.745 (0.697-0.794)	—	—	—
	Base model + standardized SII	0.750 (0.702-0.798)	0.005 (-0.004-0.018) P/q = 0.236/0.428	5.615 (1) P/q = 0.018/0.047	0.0080 (-0.0043-0.0217) P/q = 0.124/0.220
	Base model + standardized dNLR	0.745 (0.697-0.794)	0.000 (-0.004-0.009) P/q = 0.829/0.982	1.729 (1) P/q = 0.189/0.201	0.0029 (-0.0019-0.0159) P/q = 0.300/0.343
	Base model + standardized PIV	0.744 (0.695-0.792)	-0.002 (-0.008-0.011) P/q = 0.975/0.995	5.265 (1) P/q = 0.022/0.0497	0.0028 (-0.0036-0.0133) P/q = 0.513/0.548
	Base model + standardized PLR	0.758 (0.711-0.805)	0.013 (0.001-0.029) P/q = 0.022/0.117	10.051 (1) P/q = 0.002/0.012	0.0157 (-0.0030-0.0361) P/q = 0.076/0.220
	Base model + standardized CLR	0.745 (0.697-0.794)	0.000 (-0.005-0.007) P/q = 0.859/0.982	0.175 (1) P/q = 0.676/0.676	-0.0001 (-0.0017-0.0074) P/q = 1.000/1.000
	Base model + standardized LHR	0.748 (0.700-0.797)	0.003 (-0.004-0.018) P/q = 0.412/0.599	3.227 (1) P/q = 0.072/0.105	0.0066 (-0.0028-0.0242) P/q = 0.240/0.325

The base model included SOFA, APACHE II, SAPS II, and OASIS. Each marker-augmented model added one standardized marker separately. ΔAUC was calculated as the marker-augmented-model AUC minus the base-model AUC; its 95% CI and P value were estimated using 1,000 paired bootstrap samples. IDI estimates were obtained using 1,000 perturbation-resampling iterations. For each performance measure, q values were calculated using the Benjamini-Hochberg procedure across the 16 marker-outcome comparisons. All estimates are apparent in-sample estimates and may be optimistic because marker selection and performance assessment were conducted in the same cohort without optimism correction or external validation. AUC, area under the curve; CI, confidence interval; IDI, integrated discrimination improvement; LR, likelihood ratio; SOFA, Sequential Organ Failure Assessment; APACHE II, Acute Physiology and Chronic Health Evaluation II; SAPS II, Simplified Acute Physiology Score II; OASIS, Oxford Acute Severity of Illness Score; SII, systemic immune-inflammation index; NLR, neutrophil-to-lymphocyte ratio; dNLR, derived neutrophil-to-lymphocyte ratio; MLR, monocyte-to-lymphocyte ratio; NMLR, neutrophil-plus-monocyte-to-lymphocyte ratio; PIV, pan-immune-inflammation value; PLR, platelet-to-lymphocyte ratio; CLR, C-reactive protein-to-lymphocyte ratio; LHR, lymphocyte-to-high-density lipoprotein cholesterol ratio; PHR, platelet-to-high-density lipoprotein cholesterol ratio.

Global Schoenfeld-residual tests indicated departures from the proportional-hazards assumption for the severity-score base models for both 28-day mortality (P = 0.037) and 1-year mortality (P = 0.013). In the base models, non-proportionality was observed for SAPS II and OASIS at 28 days and for SOFA and OASIS at 1 year. Among the added markers, NLR, dNLR, and NMLR showed evidence of non-proportionality for 28-day mortality, whereas CLR showed evidence of non-proportionality for 1-year mortality. The nested-model results should therefore be interpreted as exploratory apparent performance estimates.

### 3.7 Multiple-testing sensitivity analysis

To assess the influence of multiple testing, the Benjamini–Hochberg procedure was applied jointly to the 36 standardized multivariable marker–outcome tests reported in **Table 2**. For 28-day mortality, standardized SII (q=0.024), NLR (q=0.043), NMLR (q=0.043), PIV (q=0.014), PLR (q=0.003), CLR (q=0.003), and LHR (q=0.026) remained statistically significant after FDR correction. In contrast, the associations of dNLR (q=0.056), MLR (q=0.088), and PHR (q=0.084) did not meet the FDR-adjusted significance threshold and were considered exploratory. For 1-year mortality, standardized PLR (q=0.009), CLR (q=0.007), and LHR (q=0.043) remained statistically significant, whereas SII (q=0.088), dNLR (q=0.088), and PIV (q=0.086) did not remain significant after FDR correction. The complete FDR-adjusted results are presented in **Supplementary Table S5**.

### 3.8 Parsimonious-model sensitivity analysis

In the primary marker-specific models reported in **Table 2**, the maximum VIF was 2.831 and the minimum tolerance was 0.353; therefore, none met the predefined diagnostic criteria for multicollinearity (**Supplementary Table S6**). In the parsimonious models adjusted for age, sex, SOFA score, and lactate, the maximum VIF was 1.137 and the minimum tolerance was 0.879 (**Supplementary Table S7**). All models included 396 patients, with 174 deaths within 28 days and 230 deaths within 1 year.

After FDR correction across the 36 parsimonious marker–outcome tests, SII (HR 1.161, 95% CI 1.043–1.291; q=0.043), PIV (HR 1.258, 95% CI 1.109–1.427; q=0.004), PLR (HR 1.299, 95% CI 1.151–1.464; q<0.001), and CLR (HR 1.269, 95% CI 1.112–1.447; q=0.004) remained associated with 28-day mortality. For 1-year mortality, PIV (HR 1.209, 95% CI 1.053–1.389; q=0.043), PLR (HR 1.277, 95% CI 1.137–1.434; q<0.001), and CLR (HR 1.193, 95% CI 1.046–1.361; q=0.043) remained associated after correction.

Although the global proportional-hazards tests were significant in all 36 parsimonious models, this did not indicate that every inflammatory–immune marker violated the proportional-hazards assumption. The global departures were driven predominantly by the shared adjustment covariates: age and lactate in the 28-day models, and age, sex, SOFA score, and lactate in the 1-year models.

The marker-specific proportional-hazards assumption was evaluated using marker × log(time) interaction terms (**Supplementary Table S7**). None of the 18 markers showed evidence of non-proportionality for 28-day mortality (all P>0.05; range, 0.070–0.983). For 1-year mortality, 15 markers satisfied the marker-specific proportional-hazards assumption, whereas SIRI, CAR, and CLR showed evidence of time-varying effects (P = 0.021, P = 0.021, and P = 0.027, respectively). Therefore, the constant-coefficient 1-year estimates for these three markers should be interpreted as overall summary associations averaged across follow-up rather than effects that remained constant over time.

### 3.9 Hospital-discharge landmark sensitivity analysis

Among the 316 patients discharged alive from the index hospitalization, 101 died after hospital discharge but before day 28 following ICU admission, and 150 died after discharge within 1 year. In this hospital-discharge landmark analysis, patients entered the risk set at hospital discharge, and only those discharged alive before the corresponding administrative endpoint contributed post-discharge risk time. For the 28-day endpoint, higher standardized MLR (HR 1.164, 95% CI 1.009–1.342; P = 0.037), SIRI (HR 1.199, 95% CI 1.045–1.374; P = 0.009), PIV (HR 1.184, 95% CI 1.035–1.354; P = 0.014), PLR (HR 1.201, 95% CI 1.043–1.383; P = 0.011), and CLR (HR 1.292, 95% CI 1.048–1.592; P = 0.016) were associated with higher post-discharge mortality before day 28. For the 1-year endpoint, most associations were attenuated after the exclusion of in-hospital deaths, whereas PLR (HR 1.180, 95% CI 1.032–1.350; P = 0.016) and CLR (HR 1.263, 95% CI 1.060–1.505; P = 0.009) remained associated with post-discharge mortality. Given the *post hoc* design and the absence of multiplicity correction, these findings should be regarded as exploratory (**Supplementary Table S8**).

### 3.10 Sensitivity analysis excluding 48-h organ-support covariates

After the 48-h organ-support covariates included in the corresponding primary models were excluded—mechanical ventilation and CRRT from the 28-day models and mechanical ventilation from the 1-year models—the principal associations of CLR, PLR, and LHR remained similar in direction and magnitude (**Supplementary Table S9**). For 28-day mortality, CLR (HR 1.288, 95% CI 1.126–1.473; P<0.001) and PLR (HR 1.273, 95% CI 1.134–1.428; P<0.001) remained positively associated with mortality, whereas LHR remained inversely associated (HR 0.754, 95% CI 0.627–0.908; P = 0.003). For 1-year mortality, the corresponding estimates were 1.261 (95% CI 1.108–1.436; P<0.001) for CLR, 1.197 (95% CI 1.075–1.332; P = 0.001) for PLR, and 0.828 (95% CI 0.713–0.961; P = 0.013) for LHR. SII, PIV, and PHR also remained associated with 28-day mortality, whereas SII, dNLR, and PIV remained associated with 1-year mortality. However, the previously observed 28-day associations of NLR, dNLR, MLR, and NMLR were attenuated and were no longer statistically significant after exclusion of the 48-h organ-support covariates. These results indicate that the principal CLR, PLR, and LHR findings were not explained solely by adjustment for mechanical ventilation or CRRT, although several short-term cellular inflammatory indices were sensitive to the covariate specification.

### 3.11 Time-varying associations with 1-year mortality

Extended Cox models were fitted for SIRI, CAR, and CLR because these markers showed evidence of marker-specific non-proportionality for 1-year mortality. The adjusted HR(t) curves showed decreasing point estimates over time, with substantial uncertainty near the extremes of follow-up (**Supplementary Figure S1**). In the complementary period-specific analysis, CLR was associated with higher mortality during days 1–90 (HR 1.222 per 1-SD increase, 95% CI 1.068–1.397; P = 0.003) but not during days 91–365 (HR 0.876, 95% CI 0.476–1.609; P = 0.669). Neither SIRI nor CAR was significantly associated with mortality in either interval: the respective HRs were 1.143 (95% CI 0.977–1.338; P = 0.095) and 0.920 (95% CI 0.790–1.072; P = 0.287) during days 1–90, and 0.585 (95% CI 0.247–1.386; P = 0.223) and 0.840 (95% CI 0.549–1.286; P = 0.422) during days 91–365 (**Supplementary Table S10**). These findings suggest that the 1-year CLR association was concentrated mainly in the early follow-up period, whereas the period-specific estimates for SIRI and CAR remained imprecise.

## 4 Discussion

In this single-center retrospective cohort of 396 sepsis patients, multivariable Cox regression identified CLR and PLR as positively associated and LHR as inversely associated with both 28-day and 1-year all-cause mortality. Most formal interaction tests did not indicate heterogeneity by age, sex, or SA-AKI status, although nominal PIV interactions were observed. RCS analyses supported nonlinear exposure–response patterns for PLR and CLR at both endpoints and an approximately linear inverse association for LHR at 28 days. These results identify adjusted associations but do not by themselves establish incremental predictive performance beyond conventional severity scores. Sensitivity analyses showed that several findings varied with parameterization and covariate adjustment; CLR and PLR were the most consistently supported across the primary, parsimonious, multiplicity-adjusted, and post-discharge landmark analyses. These primary findings were supported by a multiple-testing sensitivity analysis using the Benjamini-Hochberg procedure. Other findings varied according to the mortality endpoint or marker parameterization and should therefore be regarded as exploratory. Although some markers improved overall model fit when added individually to conventional severity scores, none produced an FDR-significant improvement in time-dependent AUC or IDI. Given the absence of internal or external validation, these findings require independent confirmation before the indices can be considered for clinical risk prediction or routine clinical use.

### 4.1 Cellular inflammation markers

The short-term associations observed for SII, NLR, NMLR, PIV, and PLR are biologically plausible in the context of sepsis. Neutrophil activation and extracellular-trap formation contribute to endothelial injury and microvascular thrombosis, whereas lymphocyte depletion reflects sepsis-induced immune dysfunction. Monocytes and platelets additionally participate in cytokine production, leukocyte recruitment, coagulation, and thromboinflammation (13, 15, 16, 52–55). Composite indices derived from these cell populations may therefore reflect several interacting components of the host response. Among these indices, PLR showed the most consistent association because it remained associated with both mortality endpoints after FDR correction. This finding is consistent with previous studies suggesting that an elevated PLR identifies an imbalance between platelet-mediated inflammation and impaired lymphocyte-dependent immunity (24, 56, 57). In contrast, SII, NLR, NMLR, and PIV retained significant associations mainly with 28-day mortality. These indices may consequently reflect the severity of acute inflammatory and immune disturbances more strongly than the determinants of long-term mortality. Their lack of consistent associations across both endpoints also supports cautious interpretation rather than ranking them as established prognostic biomarkers.

### 4.2 Inflammation-nutrition markers

CLR was one of the most robust markers in the present study. It combines CRP, an acute-phase inflammatory protein, with lymphocyte count, a measure affected by immune suppression. A high CLR may therefore identify the coexistence of systemic inflammation and impaired adaptive immunity, both of which contribute to adverse outcomes in sepsis (26, 46, 58, 59). The association of CLR with both mortality endpoints remained statistically significant after FDR correction, supporting further evaluation of this marker in independent populations. By comparison, the findings for CAR, CALLY, and HALP were less consistent. These markers incorporate CRP, albumin, hemoglobin, lymphocytes, or platelets and may reflect inflammation, nutritional status, anemia, and immune dysfunction (27, 28, 48, 60). Their performance may be influenced by hypoalbuminemia, fluid administration, renal dysfunction, chronic disease, and the timing of laboratory measurement. Moreover, several of these indices share mathematical components with each other and with variables included in the multivariable models. Although CRP was excluded as a covariate from models of CRP-containing indices to avoid mathematical coupling, overlap among their biological components may still contribute to correlated or unstable associations. These results do not exclude the potential value of CAR, CALLY, or HALP, but indicate that their prognostic relevance was less robust in this cohort.

### 4.3 HDL-C–related markers

LHR was inversely associated with both 28-day and 1-year mortality, and both associations remained statistically significant after FDR correction. By contrast, the association between PHR and 28-day mortality did not remain significant after correction, while NHR and MHR showed no consistent independent associations.

HDL-C has anti-inflammatory, antioxidant, endothelial-protective, and lipopolysaccharide-binding properties and may contribute to innate immune regulation during sepsis (50, 51, 61, 62). LHR may reflect the balance between lymphocyte-mediated host defense and HDL-C-related metabolic and endothelial functions. Nevertheless, the biological interpretation of a ratio depends on the behavior of both its numerator and denominator. The inverse association observed for LHR should therefore not be interpreted as evidence that modifying either lymphocyte count or HDL-C would improve outcomes. The underlying biological mechanism remains speculative and requires analyses that examine the individual components, dynamic changes, and relevant treatment exposures.

### 4.4 Clinical interpretation and exploratory analyses

The routinely derived inflammatory-immune indices evaluated here should be interpreted within the broader landscape of host-response assessment in sepsis. Unlike cellular immune phenotyping, biophysical leukocyte-response measurements, transcriptomic or protein-based assays, and ex vivo functional immune tests, these indices can be calculated from commonly available laboratory data and may be less resource intensive, but they are also less mechanistically specific (29). They should therefore be regarded as potential low-cost adjuncts rather than replacements for specialized host-response assays. Future prospective studies should determine whether combining these approaches improves validated discrimination, calibration, and clinical utility.

The restricted cubic spline analyses suggested nonlinear associations of log_10_-transformed PLR and CLR with both mortality endpoints, whereas the inverse association between log_10_-transformed LHR and 28-day mortality was approximately linear. These findings suggest that a single linear hazard ratio may not fully characterize the associations of PLR and CLR across their observed ranges. However, estimates at the extremes were less precise because fewer patients contributed information in these regions. The spline findings should therefore be interpreted as exploratory descriptions of exposure–response patterns rather than evidence for clinically applicable thresholds.

The nested-model analyses provided a direct assessment of whether individual markers added prognostic information beyond conventional severity scores. Some markers improved overall model fit according to likelihood-ratio tests after FDR correction. However, none of the corresponding changes in time-dependent AUC or IDI remained statistically significant after FDR correction. Improvement in model fit does not necessarily translate into clinically meaningful improvement in discrimination. Furthermore, marker selection and model-performance evaluation were conducted in the same cohort, so the reported estimates represent apparent in-sample performance and may be optimistic. Consequently, these analyses do not establish clinical utility beyond SOFA, APACHE II, SAPS II, and OASIS.

The parsimonious sensitivity analysis provided partial support for the primary findings while also demonstrating sensitivity to the choice of adjustment strategy. PLR remained FDR-significant for both mortality endpoints and did not show marker-specific departures from the proportional-hazards assumption. PIV was associated with both endpoints in the parsimonious models, although its 1-year association had not remained significant after FDR correction in the primary adjustment model. CLR remained associated with both endpoints, but its marker-specific proportional-hazards assumption was violated for 1-year mortality. Conversely, the associations of LHR observed in the primary models were not retained in the parsimonious models. These differences indicate that the estimated associations of some indices depend on the covariate set and should not be interpreted as definitive evidence that they are independent prognostic predictors.

The sensitivity analysis excluding the 48-h organ-support covariates included in the corresponding primary models provided additional information regarding the temporal definition of the adjustment variables. Mechanical ventilation and CRRT were excluded from the 28-day models, whereas only mechanical ventilation was excluded from the 1-year models because CRRT was not included in the original 1-year adjustment model. The estimates for CLR, PLR, and LHR remained similar for both mortality endpoints, suggesting that these principal associations were not driven solely by adjustment for early organ-support interventions. However, the 28-day associations of NLR, dNLR, MLR, and NMLR were attenuated and were no longer statistically significant. Thus, although this sensitivity analysis supports the robustness of the principal CLR, PLR, and LHR findings, it also indicates that some short-term cellular inflammatory indices were sensitive to the choice and timing of the adjustment covariates.

Most subgroup interaction tests did not indicate substantial heterogeneity by age, sex, or SA-AKI status. Interactions were observed between PIV and age for both mortality endpoints and between PIV and SA-AKI status for 1-year mortality. Nevertheless, these interaction analyses were exploratory, were not adjusted for multiple testing, and included relatively small samples in some strata. In particular, the directionally different PIV estimates across age groups should not be interpreted as evidence that PIV is protective in older patients. These observations require confirmation in studies specifically powered to evaluate effect modification.

The 1-year mortality findings also require additional caution. All indices were measured during the first 24 h after ICU admission and cannot be assumed to exert a sustained biological effect throughout the subsequent year. Long-term mortality is likely influenced by comorbidities, frailty, recurrent infection, rehabilitation, and post-discharge treatment, which were not fully captured. Although the global proportional-hazards tests were significant in all 36 parsimonious models, the departures were driven predominantly by shared adjustment covariates rather than by the inflammatory–immune markers themselves. Age and lactate showed time-varying effects in the 28-day models, whereas age, sex, SOFA score, and lactate showed time-varying effects in the 1-year models. This pattern is clinically plausible because the prognostic contributions of acute illness severity and host-related factors may change during follow-up. The marker-specific proportional-hazards analyses further refined the interpretation of the long-term findings. In the parsimonious sensitivity models adjusted for age, sex, SOFA score, and lactate, all 18 markers satisfied the marker-specific proportional-hazards assumption for 28-day mortality, whereas SIRI, CAR, and CLR showed evidence of time-varying associations with 1-year mortality. In the extended Cox analysis, the CLR association was evident during the first 90 days but attenuated thereafter. Therefore, the constant-coefficient 1-year HR for CLR should be interpreted as an average association over the complete follow-up period rather than a constant long-term effect. Although the time-varying point estimates for SIRI and CAR also decreased over time, their period-specific confidence intervals included unity. The wider confidence intervals during days 91–365 also indicate limited precision in the later follow-up period.

Baseline CRP and PCT concentrations and their inverse univariable associations also warrant cautious interpretation. Potential explanations include variation in sampling time relative to sepsis onset, pre-ICU antimicrobial treatment, a late immunosuppressive or immune-paralysis phenotype, survivor bias, and unmeasured differences in infection source. A sensitivity analysis excluding patients who had received antibiotics before ICU admission could not be performed because pre-ICU medication histories were incompletely recorded. The available data therefore cannot distinguish among these possible mechanisms, and the counterintuitive findings should not be interpreted as evidence that lower CRP or PCT has a causal adverse effect.

### 4.5 Strengths and limitations

This study has several strengths. First, we focused on a high-risk population of sepsis patients admitted to the ICU, which enhances the clinical relevance and practical value of our findings. Second, this study evaluated a broad panel of 18 inflammatory-immune indices for both short- and long-term mortality within the same cohort, enabling internally consistent comparisons across markers. Third, we employed restricted cubic spline analyses to reveal potential non-linear relationships between these markers and mortality risk, offering additional insights beyond simple linear assumptions. However, several limitations must be acknowledged. First, this was a single-center retrospective, hypothesis-generating study without external validation, which limits generalizability and may have reduced statistical power for weaker associations and subgroup interactions. Selection bias and information bias inherent to retrospective data collection cannot be excluded. Second, the observational design permits the identification of associations but not causal relationships. Residual confounding may remain because potentially important variables, including infection source, timing and adequacy of antimicrobial therapy, vasopressor exposure, fluid management, and frailty, were not fully available. Third, mechanical ventilation and CRRT were ascertained within 48 h, whereas the biomarker landmark was defined at 24 h. This difference in assessment windows may introduce temporal ambiguity because organ-support interventions could occur after biomarker measurement. Excluding the 48-h organ-support covariates included in the corresponding primary models—mechanical ventilation and CRRT from the 28-day models and mechanical ventilation from the 1-year models—broadly preserved the principal CLR, PLR, and LHR associations. Nevertheless, this sensitivity analysis cannot completely eliminate potential temporal or treatment-related bias. Fourth, only all-cause mortality was assessed; secondary infections, progression of organ failure, length of stay, functional status, and cause-specific mortality were not examined. Fifth, evaluating 18 markers using multiple parameterizations for two endpoints generated numerous statistical comparisons. Although the Benjamini–Hochberg procedure was applied to the 36 standardized marker–outcome tests, FDR correction reduces but does not eliminate the possibility of false-positive findings. The alternative parameterizations and subgroup interaction tests generated additional secondary comparisons and should be regarded as exploratory. Finally, the markers evaluated in the incremental prognostic analyses were selected and assessed in the same cohort, without bootstrap optimism correction, cross-validation, temporal validation, or external validation. In addition, departures from the proportional-hazards assumption were observed in the severity-score base models and some marker-augmented models. Accordingly, the likelihood-ratio, time-dependent AUC, and IDI results represent apparent in-sample performance and cannot establish clinical usefulness. The significant global proportional-hazards tests in the parsimonious models were driven predominantly by shared clinical covariates rather than by generalized non-proportionality of the inflammatory–immune markers. Marker-specific non-proportionality was identified for SIRI, CAR, and CLR in the 1-year parsimonious models. Extended Cox and period-specific analyses were performed to characterize these time-varying associations. Nevertheless, these analyses were *post hoc*, the functional form of the time interaction and the 90-day division involve modeling choices, and estimates during later follow-up were relatively imprecise. The constant-coefficient HRs for these markers should therefore be interpreted as follow-up-averaged summary associations.

## 5 Conclusion

In this exploratory single-center cohort, several composite inflammatory–immune indices showed associations with 28-day or 1-year mortality in sepsis patients. After FDR correction of the 36 standardized marker–outcome tests, SII, NLR, NMLR, PIV, PLR, CLR, and LHR remained associated with 28-day mortality, whereas PLR, CLR, and LHR remained associated with 1-year mortality. Although adding selected markers improved overall model fit in some likelihood ratio tests, none significantly improved time-dependent AUC or IDI after FDR correction. PLR showed the most consistent association across mortality endpoints and adjustment strategies. CLR also remained associated in the parsimonious analyses, although the extended Cox analysis indicated that its association with 1-year mortality was concentrated mainly within the first 90 days and attenuated during subsequent follow-up. Associations involving PIV and LHR varied according to the adjustment strategy. Given the large number of secondary comparisons and the absence of internal or external validation, these findings require independent confirmation before the indices can be considered for clinical risk prediction or routine clinical use.

## Data Availability

The raw data supporting the conclusions of this article will be made available by the authors, without undue reservation.
