# Supplementary material for: Exploratory associations of composite inflammatory-immune markers with 28-day and 1-year all-cause mortality in patients with sepsis: a retrospective cohort study
Source: Front Endocrinol (Lausanne). 2026 Oct 28;17:1946659. doi: 10.3389/fendo.2026.1946659 (PMC13752180; doi:10.3389/fendo.2026.1946659)
Supplement: Supplementary file 1 [file Supplementaryfile1.docx]

**Supplementary Table S1.** Clinical characteristics stratified by all-cause mortality

| Variables | Total population | All-cause mortality_28-day_ | | | All-cause mortality_1-year_ | | |
| --- | --- | --- | --- | --- | --- | --- | --- |
|  |  | No | Yes | P value | No | Yes | P value |
| SBP, mmHg | 122.00 (105.00, 143.25) | 124.00 (109.00, 143.00) | 121.00 (103.00, 145.00) | 0.542 | 121.00 (105.75, 138.00) | 123.00 (105.25, 146.75) | 0.300 |
| DBP, mmHg | 67.00 (57.75, 80.25) | 69.00 (59.00, 82.00) | 65.00 (55.00, 76.00) | 0.010 | 69.00 (59.00, 82.75) | 65.50 (56.00, 78.00) | 0.035 |
| HR, bpm | 103.50 (86.00, 118.25) | 100.00 (84.00, 116.00) | 105.00 (88.25, 118.75) | 0.069 | 100.00 (83.25, 119.00) | 105.00 (87.00, 118.00) | 0.203 |
| Mean arterial pressure, mmHg | 85.00 (73.00, 98.00) | 87.00 (74.00, 99.75) | 82.00 (70.25, 95.75) | 0.034 | 87.00 (74.00, 99.00) | 83.00 (73.00, 97.00) | 0.173 |
| ICU urine output_24h_, mL | 1370.00 (873.75, 2022.50) | 1567.50 (1042.50, 2280.00) | 1277.50 (732.50, 1785.00) | <0.001 | 1665.00 (1067.50, 2443.75) | 1285.00 (742.50, 1793.75) | <0.001 |
| Arterial oxygen saturation, % | 97.26 (94.97, 98.97) | 97.44 (95.54, 98.97) | 96.97 (93.36, 98.97) | 0.093 | 97.38 (95.36, 98.96) | 97.21 (94.29, 98.98) | 0.540 |
| PaO_2_, mmHg | 83.27 (69.89, 113.67) | 83.38 (72.29, 114.00) | 83.10 (67.52, 112.66) | 0.176 | 83.28 (72.23, 117.47) | 83.27 (68.53, 112.00) | 0.330 |
| PaCO_2_, mmHg | 36.89 (30.82, 45.01) | 36.09 (30.48, 44.05) | 38.16 (31.33, 46.05) | 0.094 | 36.00 (30.32, 43.52) | 37.59 (31.84, 46.10) | 0.056 |
| Ph | 7.38 (7.31, 7.42) | 7.39 (7.33, 7.42) | 7.37 (7.28, 7.43) | 0.054 | 7.38 (7.32, 7.42) | 7.38 (7.31, 7.43) | 0.798 |
| Lactate, mmol/L | 2.42 (1.51, 4.48) | 2.37 (1.47, 4.12) | 2.46 (1.64, 5.21) | 0.092 | 2.57 (1.48, 4.53) | 2.37 (1.52, 4.45) | 0.863 |
| Serum potassium, mmol/L | 4.00 (3.60, 4.40) | 4.00 (3.52, 4.30) | 4.10 (3.60, 4.50) | 0.140 | 3.90 (3.50, 4.30) | 4.10 (3.60, 4.50) | 0.021 |
| Serum sodium, mmol/L | 139.00 (135.00, 143.00) | 138.00 (135.00, 142.00) | 139.00 (135.00, 144.00) | 0.250 | 138.00 (134.00, 142.00) | 139.00 (135.00, 144.00) | 0.121 |
| Serum calcium, mmol/L | 1.95 (1.83, 2.07) | 1.96 (1.84, 2.08) | 1.92 (1.80, 2.06) | 0.141 | 1.93 (1.83, 2.06) | 1.96 (1.83, 2.08) | 0.401 |
| WBC, ×10^9^/L | 11.50 (7.25, 17.79) | 11.07 (6.93, 18.01) | 12.36 (7.96, 17.29) | 0.718 | 10.95 (6.79, 19.09) | 12.16 (7.95, 17.00) | 0.894 |
| Neutrophil count, ×10^9^/L | 10.25 (6.05, 16.15) | 9.38 (5.62, 16.20) | 10.92 (6.72, 15.56) | 0.469 | 9.46 (5.67, 17.35) | 10.46 (6.33, 15.02) | 0.878 |
| Lymphocyte count, ×10^9^/L | 0.67 (0.39, 1.07) | 0.70 (0.44, 1.16) | 0.57 (0.27, 1.01) | 0.001 | 0.69 (0.44, 1.07) | 0.64 (0.33, 1.07) | 0.131 |
| Monocyte count, ×10^9^/L | 0.30 (0.15, 0.57) | 0.34 (0.16, 0.59) | 0.27 (0.13, 0.56) | 0.178 | 0.29 (0.16, 0.59) | 0.30 (0.15, 0.56) | 0.983 |
| Hemoglobin, g/L | 109.00 (89.75, 124.00) | 110.00 (93.25, 124.75) | 107.50 (85.25, 123.75) | 0.254 | 111.00 (96.00, 125.00) | 106.00 (85.00, 123.00) | 0.054 |
| Platelet count, ×10^9^/L | 170.00 (99.75, 238.25) | 181.50 (103.50, 255.50) | 159.00 (93.00, 219.50) | 0.035 | 169.50 (100.50, 237.75) | 170.00 (98.50, 238.50) | 0.577 |
| Uric acid, μmol/L | 279.30 (200.60, 415.60) | 277.20 (211.10, 400.70) | 281.65 (179.52, 418.85) | 0.978 | 284.40 (207.60, 410.10) | 279.05 (190.88, 415.82) | 0.886 |
| Creatinine, μmol/L | 98.35 (68.92, 156.82) | 97.05 (63.55, 143.83) | 100.80 (75.38, 170.70) | 0.088 | 96.40 (64.33, 136.93) | 99.50 (72.40, 164.25) | 0.127 |
| Blood glucose, mmol/L | 8.23 (6.07, 11.30) | 8.18 (5.93, 10.80) | 8.31 (6.20, 11.37) | 0.456 | 8.21 (6.05, 10.58) | 8.27 (6.08, 11.46) | 0.587 |
| CRP, mg/L | 147.06 (61.12, 233.28) | 168.69 (68.03, 238.47) | 128.80 (55.23, 223.94) | 0.053 | 178.76 (85.00, 251.36) | 130.51 (54.65, 215.04) | 0.002 |
| PCT, ng/ml | 2.63 (0.42, 20.06) | 3.85 (0.51, 28.52) | 1.84 (0.35, 12.69) | 0.013 | 5.96 (0.83, 34.82) | 1.28 (0.32, 10.48) | <0.001 |
| Triglycerides, mmol/L | 1.37 (0.94, 2.04) | 1.30 (0.92, 2.06) | 1.43 (0.97, 1.98) | 0.375 | 1.33 (0.92, 2.23) | 1.39 (0.96, 1.96) | 0.999 |
| Total cholesterol, mmol/L | 2.68 (1.92, 3.58) | 2.61 (1.94, 3.47) | 2.79 (1.90, 3.69) | 0.344 | 2.51 (1.89, 3.38) | 2.83 (2.11, 3.71) | 0.056 |
| LDL-C, mmol/L | 1.59 (1.06, 2.20) | 1.50 (1.02, 2.11) | 1.68 (1.14, 2.33) | 0.045 | 1.45 (0.96, 2.01) | 1.69 (1.17, 2.35) | 0.002 |
| HDL-C, mmol/L | 0.70 (0.52, 0.91) | 0.71 (0.53, 0.89) | 0.68 (0.50, 0.93) | 0.795 | 0.69 (0.51, 0.88) | 0.71 (0.52, 0.92) | 0.411 |
| BNP, pg/mL | 3132.50 (857.62, 8946.00) | 2277.00 (643.90, 7592.00) | 4181.00 (1426.00, 9910.00) | <0.001 | 2075.50 (611.57, 7628.00) | 3764.50 (1308.75, 9366.75) | 0.008 |
| Troponin T, ng/mL | 0.06 (0.02, 0.16) | 0.05 (0.02, 0.12) | 0.08 (0.03, 0.20) | <0.001 | 0.04 (0.02, 0.11) | 0.07 (0.03, 0.18) | <0.001 |
| ALT, U/L | 25.95 (13.20, 55.30) | 25.70 (13.12, 51.67) | 26.20 (13.62, 57.95) | 0.370 | 26.05 (14.05, 58.83) | 25.90 (13.03, 53.38) | 0.808 |
| Total bilirubin, μmol/L | 15.75 (10.10, 25.52) | 15.35 (10.62, 24.40) | 16.10 (9.55, 26.58) | 0.990 | 16.30 (11.10, 24.40) | 15.20 (9.93, 26.50) | 0.398 |
| Albumin, g/L | 26.90 (23.90, 30.50) | 27.10 (23.92, 31.00) | 26.50 (23.55, 29.68) | 0.136 | 27.35 (24.00, 30.98) | 26.65 (23.75, 29.85) | 0.232 |
| Prothrombin time, s | 13.70 (12.40, 16.10) | 13.60 (12.30, 15.67) | 13.90 (12.60, 16.27) | 0.054 | 13.80 (12.40, 16.10) | 13.70 (12.33, 16.03) | 0.592 |
| D-dimer, mg/L | 5.15 (2.34, 10.66) | 4.63 (2.30, 9.30) | 6.01 (2.46, 13.41) | 0.174 | 5.09 (2.38, 10.05) | 5.55 (2.28, 12.30) | 0.983 |
| SII | 2237.8094 (1002.7305, 4862.0227) | 2047.9762 (987.2500, 4165.1118) | 2574.7091 (1000.7452, 5635.7462) | 0.213 | 2247.9165 (1101.3088, 4252.2885) | 2176.2500 (928.3648, 5414.5212) | 0.803 |
| NLR | 14.3912 (6.9353, 31.8209) | 12.2851 (6.2958, 30.2164) | 17.7750 (8.8312, 35.3306) | 0.014 | 13.5523 (6.8970, 30.5487) | 15.3613 (6.9106, 33.0628) | 0.508 |
| dNLR | 9.1452 (4.6478–16.4981) | 8.2181 (4.3264–16.3139) | 10.0522 (5.7959–18.8537) | 0.032 | 9.2027 (4.7224–16.8325) | 9.1452 (4.5460–16.2814) | 0.907 |
| MLR | 0.4354 (0.2335–0.8431) | 0.3941 (0.2239–0.8163) | 0.5022 (0.2611–0.9215) | 0.065 | 0.3859 (0.2056–0.7914) | 0.4810 (0.2611–0.8898) | 0.069 |
| NMLR | 14.9904 (7.2494, 32.6068) | 13.1472 (6.6457, 30.8076) | 18.5343 (9.2789, 35.9062) | 0.015 | 13.7165 (7.1030, 31.3403) | 15.8325 (7.3028, 33.5827) | 0.480 |
| SIRI | 4.9299 (1.6665, 11.5504) | 3.9287 (1.5202, 11.1127) | 5.8863 (1.8911, 12.1212) | 0.142 | 4.2810 (1.4913, 11.2723) | 5.2059 (1.7944, 11.7070) | 0.425 |
| PIV | 695.0094 (207.7041, 2067.1657) | 668.7908 (239.0123, 1927.5475) | 717.6343 (173.5294, 2282.2610) | 0.663 | 638.7157 (226.0845, 1927.5475) | 718.8753 (199.3848, 2155.1417) | 0.667 |
| PLR | 220.2311 (140.1078, 481.8338) | 203.2156 (135.1351, 391.2587) | 259.5537 (150.3150, 556.9746) | 0.028 | 203.2156 (143.0877, 383.0769) | 245.2037 (127.5510, 525.0992) | 0.174 |
| NPR | 0.0577 (0.0331, 0.1077) | 0.0527 (0.0288, 0.1012) | 0.0666 (0.0379, 0.1091) | 0.058 | 0.0552 (0.0290, 0.1165) | 0.0589 (0.0356, 0.1014) | 0.769 |
| PAR | 6.2316 (3.7464, 8.8316) | 6.5139 (3.8960, 9.5004) | 5.9329 (3.6583, 8.5332) | 0.128 | 6.4428 (3.6737, 9.2030) | 6.0369 (3.7578, 8.7352) | 0.768 |
| CAR | 5.7049 (2.3640, 9.0706) | 6.0286 (2.9219, 9.1679) | 5.1147 (1.9646, 8.8152) | 0.187 | 6.5110 (3.2441, 9.5725) | 5.2666 (1.9646, 8.2988) | 0.013 |
| CLR | 217.5625 (78.4140, 459.5087) | 208.9203 (80.4604, 428.3685) | 230.4862 (73.8175, 557.6182) | 0.338 | 224.0840 (95.3431, 460.3554) | 204.9657 (70.9537, 455.2082) | 0.427 |
| CALLY | 0.0119 (0.0052, 0.0337) | 0.0124 (0.0057, 0.0336) | 0.0111 (0.0044, 0.0343) | 0.252 | 0.0116 (0.0053, 0.0289) | 0.0125 (0.0052, 0.0402) | 0.556 |
| HALP | 11.9506 (6.0077, 22.2755) | 14.8748 (6.9113, 23.1331) | 10.3069 (4.9701, 20.6099) | 0.011 | 14.8748 (7.2253, 22.8696) | 10.5545 (5.2257, 21.7933) | 0.038 |
| NHR | 14.7491 (7.5929, 26.0902) | 13.8621 (7.4721, 23.4706) | 15.6777 (7.8686, 27.3913) | 0.562 | 14.1294 (7.5680, 26.7847) | 14.8136 (7.5775, 25.1400) | 0.592 |
| MHR | 0.4243 (0.2164, 0.8679) | 0.4629 (0.2424, 0.8607) | 0.3790 (0.1727, 0.8857) | 0.199 | 0.4150 (0.2377, 0.8607) | 0.4547 (0.1911, 0.8829) | 0.780 |
| LHR | 0.9753 (0.5392, 1.7067) | 1.1419 (0.6031, 1.7884) | 0.8700 (0.3794, 1.6045) | 0.006 | 1.0653 (0.6129, 1.6191) | 0.9043 (0.4431, 1.7940) | 0.190 |
| PHR | 229.3381 (143.9295, 371.7421) | 249.9007 (148.1389, 381.1740) | 216.4047 (142.3758, 339.2910) | 0.087 | 235.9902 (140.2049, 381.1740) | 224.7778 (147.0432, 364.2628) | 0.454 |

SBP, Systolic blood pressure_;_ DBP, Diastolic blood pressure; HR, Heart rate_;_ PaO_2_, Partial pressure of oxygen_;_ PaCO_2_, Partial pressure of carbon dioxide_;_ Ph, Potential of hydrogen; WBC, White blood cell; CRP, C-reactive protein; PCT, Procalcitonin_;_ LDL-C, Low-density lipoprotein cholesterol_;_ HDL-C, High density lipoprotein cholesterol; BNP, Brain natriuretic peptide; ALT, Alanine aminotransferase; SII, Systemic immune-inflammation index; NLR, Neutrophil-to-lymphocyte ratio; dNLR, Derived neutrophil-to-lymphocyte ratio; MLR, Monocyte-to-lymphocyte ratio; NMLR, Neutrophil plus monocyte-to-lymphocyte ratio; SIRI, Systemic inflammation response index; PIV, Pan-immune-inflammation value; PLR, Platelet-to-lymphocyte ratio; NPR, Neutrophil-to-platelet ratio; PAR, Platelet-to-albumin ratio; CAR, C-reactive protein-to-albumin ratio; CLR, C-reactive protein-to-lymphocyte ratio; CALLY, C-reactive protein-albumin-lymphocyte index; HALP, Hemoglobin-albumin-lymphocyte-platelet score; NHR, Neutrophil-to-high-density lipoprotein cholesterol ratio; MHR, Monocyte-to-high-density lipoprotein cholesterol ratio; LHR, Lymphocyte-to-high-density lipoprotein cholesterol ratio; PHR, Platelet-to-high-density lipoprotein cholesterol ratio.

**Supplementary Table S2.** Cohort median cut-offs of the 18 inflammatory-immune indices

| Index | Median cut-off | Low, n | High, n |
| --- | --- | --- | --- |
| SII | ≤2237.81 vs >2237.81 | 198 | 198 |
| NLR | ≤14.39 vs >14.39 | 198 | 198 |
| dNLR | ≤9.15 vs >9.15 | 198 | 198 |
| MLR | ≤0.44 vs >0.44 | 198 | 198 |
| NMLR | ≤14.99 vs >14.99 | 198 | 198 |
| SIRI | ≤4.93 vs >4.93 | 198 | 198 |
| PIV | ≤695.01 vs >695.01 | 198 | 198 |
| PLR | ≤220.23 vs >220.23 | 198 | 198 |
| NPR | ≤0.06 vs >0.06 | 198 | 198 |
| PAR | ≤6.23 vs >6.23 | 198 | 198 |
| CAR | ≤5.70 vs >5.70 | 198 | 198 |
| CLR | ≤217.56 vs >217.56 | 198 | 198 |
| CALLY | ≤0.01 vs >0.01 | 198 | 198 |
| HALP | ≤11.95 vs >11.95 | 198 | 198 |
| NHR | ≤14.75 vs >14.75 | 198 | 198 |
| MHR | ≤0.42 vs >0.42 | 198 | 198 |
| LHR | ≤0.98 vs >0.98 | 198 | 198 |
| PHR | ≤229.34 vs >229.34 | 198 | 198 |

SII, Systemic immune-inflammation index; NLR, Neutrophil-to-lymphocyte ratio; dNLR, Derived neutrophil-to-lymphocyte ratio; MLR, Monocyte-to-lymphocyte ratio; NMLR, Neutrophil plus monocyte-to-lymphocyte ratio; SIRI, Systemic inflammation response index; PIV, Pan-immune-inflammation value; PLR, Platelet-to-lymphocyte ratio; NPR, Neutrophil-to-platelet ratio; PAR, Platelet-to-albumin ratio; CAR, C-reactive protein-to-albumin ratio; CLR, C-reactive protein-to-lymphocyte ratio; CALLY, C-reactive protein-albumin-lymphocyte index; HALP, Hemoglobin-albumin-lymphocyte-platelet score; NHR, Neutrophil-to-high-density lipoprotein cholesterol ratio; MHR, Monocyte-to-high-density lipoprotein cholesterol ratio; LHR, Lymphocyte-to-high-density lipoprotein cholesterol ratio; PHR, Platelet-to-high-density lipoprotein cholesterol ratio.

**Supplementary Table S3.** Univariate Cox regression analysis for all-cause mortality

| Variables | All-cause mortality_28-day_ | | | All-cause mortality_1-year_ | | |
| --- | --- | --- | --- | --- | --- | --- |
|  | HR | 95% CI | P value | HR | 95% CI | P value |
| Age | 1.020 | 1.009-1.031 | <0.001 | 1.029 | 1.019-1.039 | <0.001 |
| Sex |  |  |  |  |  |  |
| Male | Ref |  |  | Ref |  |  |
| Female | 1.046 | 0.769-1.423 | 0.775 | 0.902 | 0.687-1.186 | 0.460 |
| SA-AKI |  |  |  |  |  |  |
| Yes | Ref |  |  | Ref |  |  |
| No | 0.756 | 0.561-1.017 | 0.065 | 0.775 | 0.598-1.004 | 0.054 |
| CHF |  |  |  |  |  |  |
| Yes | Ref |  |  | Ref |  |  |
| No | 0.698 | 0.468-1.040 | 0.077 | 0.649 | 0.458-0.920 | 0.015 |
| Cerebrovascular disease |  |  |  |  |  |  |
| Yes | Ref |  |  | Ref |  |  |
| No | 0.966 | 0.715-1.307 | 0.825 | 0.792 | 0.610-1.027 | 0.078 |
| Diabetes |  |  |  |  |  |  |
| Yes | Ref |  |  | Ref |  |  |
| No | 0.902 | 0.654-1.243 | 0.527 | 0.888 | 0.671-1.175 | 0.405 |
| ARDS |  |  |  |  |  |  |
| Yes | Ref |  |  | Ref |  |  |
| No | 1.097 | 0.646-1.863 | 0.731 | 1.082 | 0.684-1.712 | 0.735 |
| SBP | 0.997 | 0.992-1.002 | 0.252 | 1.001 | 0.996-1.005 | 0.786 |
| DBP | 0.988 | 0.979-0.997 | 0.013 | 0.990 | 0.982-0.998 | 0.016 |
| HR | 1.007 | 1.000-1.013 | 0.040 | 1.005 | 0.999-1.010 | 0.092 |
| Mean arterial pressure | 0.990 | 0.982-0.999 | 0.022 | 0.993 | 0.986-1.001 | 0.070 |
| ICU urine output_24h_ | 1.000 | 0.999-1.000 | <0.001 | 1.000 | 0.999-1.000 | <0.001 |
| Arterial oxygen saturation | 0.963 | 0.945-0.983 | <0.001 | 0.969 | 0.950-0.988 | 0.001 |
| PaO_2_ | 1.001 | 0.997-1.004 | 0.671 | 1.000 | 0.997-1.003 | 0.863 |
| PaCO_2_ | 1.010 | 1.001-1.020 | 0.027 | 1.009 | 1.001-1.017 | 0.026 |
| Ph | 0.076 | 0.016-0.356 | 0.001 | 0.276 | 0.066-1.154 | 0.078 |
| Lactate | 1.077 | 1.029-1.127 | 0.001 | 1.046 | 1.002-1.093 | 0.039 |
| Serum potassium | 1.332 | 1.053-1.686 | 0.017 | 1.369 | 1.113-1.684 | 0.003 |
| Serum sodium | 1.012 | 0.992-1.033 | 0.230 | 1.016 | 0.998-1.033 | 0.078 |
| Serum calcium | 0.635 | 0.327-1.235 | 0.181 | 0.935 | 0.517-1.692 | 0.824 |
| WBC | 1.000 | 0.984-1.017 | 0.982 | 0.995 | 0.981-1.010 | 0.515 |
| Neutrophil count | 1.002 | 0.985-1.019 | 0.849 | 0.995 | 0.980-1.010 | 0.510 |
| Lymphocyte count | 0.777 | 0.605-0.999 | 0.049 | 0.953 | 0.784-1.159 | 0.631 |
| Monocyte count | 1.068 | 0.676-1.688 | 0.777 | 1.131 | 0.765-1.673 | 0.538 |
| Hemoglobin | 0.997 | 0.991-1.004 | 0.422 | 0.996 | 0.990-1.001 | 0.125 |
| Platelet count | 0.998 | 0.997-1.000 | 0.041 | 0.999 | 0.998-1.001 | 0.266 |
| Uric acid | 1.000 | 1.000-1.001 | 0.307 | 1.000 | 1.000-1.001 | 0.384 |
| Creatinine | 1.000 | 0.999-1.001 | 0.418 | 1.000 | 1.000-1.001 | 0.416 |
| Blood glucose | 1.023 | 0.991-1.056 | 0.163 | 1.027 | 1.000-1.056 | 0.053 |
| CRP | 0.999 | 0.998-1.001 | 0.284 | 0.998 | 0.997-1.000 | 0.028 |
| PCT | 0.993 | 0.986-0.999 | 0.034 | 0.988 | 0.981-0.994 | <0.001 |
| Triglycerides | 1.082 | 0.953-1.227 | 0.223 | 1.015 | 0.903-1.141 | 0.806 |
| Total cholesterol | 1.039 | 0.925-1.167 | 0.522 | 1.059 | 0.960-1.169 | 0.253 |
| LDL-C | 1.138 | 0.973-1.331 | 0.106 | 1.164 | 1.020-1.329 | 0.024 |
| HDL-C | 0.945 | 0.561-1.592 | 0.833 | 1.079 | 0.687-1.696 | 0.740 |
| BNP | 1.000 | 1.000-1.000 | 0.009 | 1.000 | 1.000-1.000 | 0.022 |
| Troponin T | 0.996 | 0.984-1.008 | 0.528 | 0.995 | 0.984-1.007 | 0.451 |
| ALT | 1.001 | 1.001-1.001 | <0.001 | 1.001 | 1.001-1.001 | <0.001 |
| Total bilirubin | 0.999 | 0.995-1.004 | 0.815 | 1.000 | 0.996-1.003 | 0.949 |
| Albumin | 0.969 | 0.939-0.999 | 0.046 | 0.976 | 0.950-1.002 | 0.076 |
| Prothrombin time | 1.040 | 1.011-1.069 | 0.007 | 1.028 | 0.998-1.059 | 0.065 |
| D-dimer | 1.011 | 1.003-1.019 | 0.007 | 1.006 | 0.998-1.014 | 0.141 |
| SOFA_24h_ | 1.086 | 1.054-1.119 | <0.001 | 1.072 | 1.043-1.103 | <0.001 |
| APACHE II_24h_ | 1.086 | 1.063-1.109 | <0.001 | 1.084 | 1.065-1.103 | <0.001 |
| GCS_24h_ | 0.943 | 0.913-0.973 | <0.001 | 0.933 | 0.908-0.960 | <0.001 |
| SAPS II score_24h_ | 1.032 | 1.024-1.041 | <0.001 | 1.031 | 1.024-1.039 | <0.001 |
| SIRS_24h_ | 1.204 | 1.032-1.404 | 0.018 | 1.128 | 0.988-1.287 | 0.075 |
| OASIS_24h_ | 1.050 | 1.030-1.070 | <0.001 | 1.047 | 1.030-1.065 | <0.001 |
| Mechanical ventilation_48h_ |  |  |  |  |  |  |
| Yes | Ref |  |  | Ref |  |  |
| No | 0.279 | 0.137-0.568 | <0.001 | 0.350 | 0.204-0.601 | <0.001 |
| CRRT_48h_ |  |  |  |  |  |  |
| Yes | Ref |  |  | Ref |  |  |
| No | 0.575 | 0.404-0.819 | 0.002 | 0.722 | 0.520-1.004 | 0.053 |
| SII | 1.000 | 1.000-1.000 | 0.003 | 1.000 | 1.000-1.000 | 0.010 |
| Standardized SII | 1.166 | 1.052-1.292 | 0.003 | 1.147 | 1.034-1.273 | 0.010 |
| NLR | 1.006 | 1.002-1.010 | 0.002 | 1.005 | 1.001-1.009 | 0.023 |
| Standardized NLR | 1.177 | 1.059-1.308 | 0.002 | 1.138 | 1.018-1.272 | 0.023 |
| dNLR | 1.013 | 1.000–1.027 | 0.052 | 1.006 | 0.994–1.020 | 0.328 |
| Standardized dNLR | 1.141 | 0.999–1.304 | 0.052 | 1.066 | 0.938–1.210 | 0.328 |
| MLR | 1.292 | 1.077–1.551 | 0.006 | 1.234 | 1.037–1.469 | 0.018 |
| Standardized MLR | 1.205 | 1.055–1.376 | 0.006 | 1.165 | 1.027–1.323 | 0.018 |
| NMLR | 1.006 | 1.002-1.009 | 0.002 | 1.005 | 1.001-1.008 | 0.021 |
| Standardized NMLR | 1.179 | 1.061-1.311 | 0.002 | 1.140 | 1.020-1.275 | 0.021 |
| SIRI | 1.008 | 1.002-1.014 | 0.007 | 1.006 | 0.999-1.012 | 0.104 |
| Standardized SIRI | 1.192 | 1.049-1.355 | 0.007 | 1.128 | 0.976-1.305 | 0.104 |
| PIV | 1.000 | 1.000-1.000 | 0.002 | 1.000 | 1.000-1.000 | 0.014 |
| Standardized PIV | 1.206 | 1.074-1.355 | 0.002 | 1.170 | 1.032-1.326 | 0.014 |
| PLR | 1.001 | 1.000-1.001 | <0.001 | 1.001 | 1.000-1.001 | <0.001 |
| Standardized PLR | 1.253 | 1.109-1.417 | <0.001 | 1.233 | 1.096-1.387 | <0.001 |
| NPR | 1.205 | 0.761-1.907 | 0.427 | 1.044 | 0.646-1.686 | 0.862 |
| Standardized NPR | 1.054 | 0.930-1.194 | 0.427 | 1.012 | 0.888-1.153 | 0.862 |
| PAR | 0.972 | 0.937-1.008 | 0.126 | 0.988 | 0.959-1.018 | 0.434 |
| Standardized PAR | 0.882 | 0.752-1.036 | 0.126 | 0.948 | 0.830-1.083 | 0.434 |
| CAR | 0.995 | 0.961-1.031 | 0.783 | 0.977 | 0.947-1.009 | 0.153 |
| Standardized CAR | 0.978 | 0.837-1.144 | 0.783 | 0.902 | 0.783-1.039 | 0.153 |
| CLR | 1.000 | 1.000-1.001 | 0.002 | 1.000 | 1.000-1.000 | 0.082 |
| Standardized CLR | 1.217 | 1.072-1.383 | 0.002 | 1.120 | 0.986-1.273 | 0.082 |
| CALLY | 0.823 | 0.433-1.566 | 0.554 | 1.029 | 0.694-1.527 | 0.885 |
| Standardized CALLY | 0.943 | 0.776-1.145 | 0.554 | 1.009 | 0.895-1.137 | 0.885 |
| HALP | 1.001 | 0.999-1.004 | 0.361 | 1.001 | 0.999-1.004 | 0.280 |
| Standardized HALP | 1.061 | 0.935-1.203 | 0.361 | 1.067 | 0.949-1.199 | 0.280 |
| NHR | 1.000 | 0.993-1.006 | 0.912 | 0.999 | 0.994-1.005 | 0.795 |
| Standardized NHR | 0.992 | 0.858-1.146 | 0.912 | 0.983 | 0.865-1.117 | 0.795 |
| MHR | 1.022 | 0.843-1.239 | 0.823 | 1.028 | 0.870-1.215 | 0.746 |
| Standardized MHR | 1.017 | 0.874-1.185 | 0.823 | 1.022 | 0.895-1.167 | 0.746 |
| LHR | 0.906 | 0.796-1.030 | 0.132 | 0.955 | 0.864-1.056 | 0.368 |
| Standardized LHR | 0.871 | 0.728-1.042 | 0.132 | 0.938 | 0.816-1.078 | 0.368 |
| PHR | 0.999 | 0.999-1.000 | 0.079 | 1.000 | 0.999-1.000 | 0.280 |
| Standardized PHR | 0.854 | 0.716-1.018 | 0.079 | 0.926 | 0.806-1.064 | 0.280 |

SA-AKI, Sepsis-associated acute kidney injury; CHF, Chronic heart failure; ARDS, Acute respiratory distress syndrome; SBP, Systolic blood pressure; DBP, Diastolic blood pressure; HR, Heart rate; PaO_2_, Partial pressure of oxygen; PaCO_2_, Partial pressure of carbon dioxide; Ph, Potential of hydrogen; WBC, White blood cell; CRP, C-reactive protein; PCT, Procalcitonin; LDL-C, Low-density lipoprotein cholesterol; HDL-C, High density lipoprotein cholesterol; BNP, Brain natriuretic peptide; ALT, Alanine aminotransferase; SOFA, Sequential organ failure assessment; APACHE II, Acute physiology and chronic health evaluation II; GCS, Glasgow coma scale; SAPS II, Simplified acute physiology score II; SIRS, Systemic inflammatory response syndrome; OASIS, Oxford acute severity of illness score; CRRT, continuous renal replacement therapy; SII, Systemic immune-inflammation index; NLR, Neutrophil-to-lymphocyte ratio; dNLR, Derived neutrophil-to-lymphocyte ratio; MLR, Monocyte-to-lymphocyte ratio; NMLR, Neutrophil plus monocyte-to-lymphocyte ratio; SIRI, Systemic inflammation response index; PIV, Pan-immune-inflammation value; PLR, Platelet-to-lymphocyte ratio; NPR, Neutrophil-to-platelet ratio; PAR, Platelet-to-albumin ratio; CAR, C-reactive protein-to-albumin ratio; CLR, C-reactive protein-to-lymphocyte ratio; CALLY, C-reactive protein-albumin-lymphocyte index; HALP, Hemoglobin-albumin-lymphocyte-platelet score; NHR, Neutrophil-to-high-density lipoprotein cholesterol ratio; MHR, Monocyte-to-high-density lipoprotein cholesterol ratio; LHR, Lymphocyte-to-high-density lipoprotein cholesterol ratio; PHR, Platelet-to-high-density lipoprotein cholesterol ratio.

**Supplementary Table S4.** Multivariate Cox regression analysis for all-cause mortality

| Variables | All-cause mortality_28-day_ | | | All-cause mortality_1-year_ | | |
| --- | --- | --- | --- | --- | --- | --- |
|  | HR | 95% CI | P value | HR | 95% CI | P value |
| **SII** | 1.000 | 1.000-1.000 | 0.004 | 1.000 | 1.000-1.000 | 0.039 |
| Log_10_SII | 1.111 | 0.840-1.470 | 0.462 | 1.098 | 0.855-1.411 | 0.463 |
| SII |  |  |  |  |  |  |
| Low SII | Ref |  |  | Ref |  |  |
| High SII | 1.417 | 1.047-1.919 | 0.024 | 1.229 | 0.932-1.621 | 0.144 |
| **NLR** | 1.005 | 1.001-1.008 | 0.012 | 1.003 | 0.999-1.007 | 0.118 |
| Log_10_NLR | 1.511 | 1.096-2.083 | 0.012 | 1.295 | 0.966-1.737 | 0.083 |
| NLR |  |  |  |  |  |  |
| Low NLR | Ref |  |  | Ref |  |  |
| High NLR | 1.495 | 1.105-2.022 | 0.009 | 1.443 | 1.108-1.879 | 0.007 |
| **dNLR** | 1.016 | 1.003–1.030 | 0.017 | 1.013 | 1.001–1.026 | 0.039 |
| Log_10_dNLR | 1.609 | 1.072–2.415 | 0.022 | 1.365 | 0.950–1.961 | 0.093 |
| dNLR |  |  |  |  |  |  |
| Low dNLR | Ref |  |  | Ref |  |  |
| High dNLR | 1.443 | 1.067–1.952 | 0.017 | 1.301 | 0.981–1.726 | 0.068 |
| **MLR** | 1.199 | 1.010–1.424 | 0.039 | 1.109 | 0.928–1.326 | 0.254 |
| Log_10_MLR | 1.462 | 1.016–2.103 | 0.041 | 1.285 | 0.921–1.791 | 0.140 |
| MLR |  |  |  |  |  |  |
| Low MLR | Ref |  |  | Ref |  |  |
| High MLR | 1.399 | 1.035–1.890 | 0.029 | 1.235 | 0.939–1.625 | 0.131 |
| **NMLR** | 1.005 | 1.001-1.008 | 0.012 | 1.003 | 0.999-1.007 | 0.118 |
| Log_10_NMLR | 1.527 | 1.102-2.115 | 0.011 | 1.336 | 1.003-1.779 | 0.047 |
| NMLR |  |  |  |  |  |  |
| Low NMLR | Ref |  |  | Ref |  |  |
| High NMLR | 1.494 | 1.105-2.022 | 0.009 | 1.437 | 1.103-1.872 | 0.007 |
| **SIRI** | 1.005 | 0.998-1.012 | 0.196 | 1.004 | 0.996-1.011 | 0.343 |
| Log_10_SIRI | 1.045 | 0.837-1.305 | 0.696 | 1.056 | 0.864-1.290 | 0.595 |
| SIRI |  |  |  |  |  |  |
| Low SIRI | Ref |  |  | Ref |  |  |
| High SIRI | 1.425 | 1.053-1.929 | 0.022 | 1.284 | 0.979-1.685 | 0.071 |
| **PIV** | 1.000 | 1.000-1.000 | 0.002 | 1.000 | 1.000-1.000 | 0.031 |
| Log_10_PIV | 0.975 | 0.804-1.184 | 0.800 | 0.993 | 0.831-1.186 | 0.938 |
| PIV |  |  |  |  |  |  |
| Low PIV | Ref |  |  | Ref |  |  |
| High PIV | 1.076 | 0.783-1.481 | 0.651 | 1.152 | 0.879-1.511 | 0.306 |
| **PLR** | 1.001 | 1.000-1.001 | <0.001 | 1.000 | 1.000-1.001 | 0.001 |
| Log_10_PLR | 1.516 | 1.041-2.206 | 0.030 | 1.336 | 0.949-1.880 | 0.097 |
| PLR |  |  |  |  |  |  |
| Low PLR | Ref |  |  | Ref |  |  |
| High PLR | 1.464 | 1.074-1.994 | 0.016 | 1.470 | 1.125-1.919 | 0.005 |
| **NPR** | 1.174 | 0.695-1.985 | 0.548 | 1.166 | 0.701-1.939 | 0.554 |
| Log_10_NPR | 1.034 | 0.731-1.463 | 0.850 | 1.054 | 0.781-1.422 | 0.733 |
| NPR |  |  |  |  |  |  |
| Low NPR | Ref |  |  | Ref |  |  |
| High NPR | 1.140 | 0.819-1.587 | 0.436 | 1.064 | 0.810-1.399 | 0.654 |
| **PAR** | 0.973 | 0.935-1.014 | 0.196 | 0.982 | 0.949-1.017 | 0.320 |
| Log_10_PAR | 0.663 | 0.398-1.104 | 0.114 | 0.717 | 0.453-1.136 | 0.156 |
| PAR |  |  |  |  |  |  |
| Low PAR | Ref |  |  | Ref |  |  |
| High PAR | 0.883 | 0.635-1.227 | 0.457 | 0.831 | 0.629-1.098 | 0.194 |
| **CAR** | 0.998 | 0.960-1.037 | 0.924 | 0.994 | 0.962-1.028 | 0.728 |
| Log_10_CAR | 0.851 | 0.599-1.210 | 0.370 | 0.906 | 0.675-1.217 | 0.514 |
| CAR |  |  |  |  |  |  |
| Low CAR | Ref |  |  | Ref |  |  |
| High CAR | 0.833 | 0.602-1.153 | 0.271 | 0.856 | 0.649-1.131 | 0.274 |
| **CLR** | 1.001 | 1.000-1.001 | <0.001 | 1.000 | 1.000-1.001 | <0.001 |
| Log_10_CLR | 1.309 | 0.960-1.785 | 0.088 | 1.204 | 0.933-1.555 | 0.153 |
| CLR |  |  |  |  |  |  |
| Low CLR | Ref |  |  | Ref |  |  |
| High CLR | 1.319 | 0.950-1.833 | 0.099 | 1.216 | 0.922-1.604 | 0.167 |
| **CALLY** | 0.967 | 0.495-1.890 | 0.921 | 1.016 | 0.642-1.608 | 0.947 |
| Log_10_CALLY | 0.775 | 0.575-1.044 | 0.094 | 0.840 | 0.657-1.074 | 0.164 |
| CALLY |  |  |  |  |  |  |
| Low CALLY | Ref |  |  | Ref |  |  |
| High CALLY | 0.863 | 0.618-1.205 | 0.387 | 0.876 | 0.663-1.158 | 0.352 |
| **HALP** | 1.001 | 0.998-1.004 | 0.483 | 1.001 | 0.999-1.004 | 0.247 |
| Log_10_HALP | 0.694 | 0.491-0.981 | 0.039 | 0.774 | 0.561-1.067 | 0.117 |
| HALP |  |  |  |  |  |  |
| Low HALP | Ref |  |  | Ref |  |  |
| High HALP | 0.642 | 0.473-0.871 | 0.004 | 0.694 | 0.534-0.904 | 0.007 |
| **NHR** | 0.994 | 0.987-1.001 | 0.072 | 0.998 | 0.992-1.003 | 0.385 |
| Log_10_NHR | 0.790 | 0.573-1.090 | 0.151 | 0.866 | 0.650-1.154 | 0.327 |
| NHR |  |  |  |  |  |  |
| Low NHR | Ref |  |  | Ref |  |  |
| High NHR | 1.006 | 0.721-1.403 | 0.973 | 1.084 | 0.826-1.423 | 0.559 |
| **MHR** | 0.882 | 0.725-1.073 | 0.211 | 0.931 | 0.779-1.111 | 0.426 |
| Log_10_MHR | 0.730 | 0.528-1.010 | 0.058 | 0.808 | 0.604-1.081 | 0.152 |
| MHR |  |  |  |  |  |  |
| Low MHR | Ref |  |  | Ref |  |  |
| High MHR | 0.718 | 0.521-0.989 | 0.043 | 0.801 | 0.606-1.059 | 0.119 |
| **LHR** | 0.820 | 0.715-0.942 | 0.005 | 0.866 | 0.776-0.966 | 0.010 |
| Log_10_LHR | 0.465 | 0.326-0.664 | <0.001 | 0.576 | 0.421-0.788 | <0.001 |
| LHR |  |  |  |  |  |  |
| Low LHR | Ref |  |  | Ref |  |  |
| High LHR | 0.628 | 0.462-0.854 | 0.003 | 0.682 | 0.523-0.890 | 0.005 |
| **PHR** | 0.999 | 0.998-1.000 | 0.028 | 1.000 | 0.999-1.000 | 0.177 |
| Log_10_PHR | 0.603 | 0.385-0.945 | 0.027 | 0.676 | 0.448-1.020 | 0.062 |
| PHR |  |  |  |  |  |  |
| Low PHR | Ref |  |  | Ref |  |  |
| High PHR | 0.678 | 0.487-0.944 | 0.021 | 0.810 | 0.615-1.068 | 0.135 |

For all-cause mortality_28-day_, multivariate Cox regression analysis adjusted for age, heart rate, mean arterial pressure, ICU urine output_24h_, arterial oxygen saturation, PaCO_2_, Ph, lactate, serum potassium, PCT, BNP, ALT, Prothrombin time, D-dimer, SOFA_24h_, APACHE II_24h_, GCS_24h_, SAPS II score_24h_, SIRS_24h_, OASIS_24h_, Mechanical ventilation_48h_, CRRT_48h_.

For all-cause mortality_1-year_, multivariate Cox regression analysis adjusted for age, ICU urine output_24h_, PaCO_2_, arterial oxygen saturation, CRP, PCT, lactate, serum potassium, ALT, BNP, SOFA_24h_, APACHE II_24h_, GCS_24h_, SAPS II score_24h_, OASIS_24h_, and mechanical ventilation_48h_. Because CRP is a component of CAR, CLR, and CALLY, the multivariable models for these three indices excluded CRP as a covariate to avoid mathematical coupling.

SII, Systemic immune-inflammation index; NLR, Neutrophil-to-lymphocyte ratio; dNLR, Derived neutrophil-to-lymphocyte ratio; MLR, Monocyte-to-lymphocyte ratio; NMLR, Neutrophil plus monocyte-to-lymphocyte ratio; SIRI, Systemic inflammation response index; PIV, Pan-immune-inflammation value; PLR, Platelet-to-lymphocyte ratio; NPR, Neutrophil-to-platelet ratio; PAR, Platelet-to-albumin ratio; CAR, C-reactive protein-to-albumin ratio; CLR, C-reactive protein-to-lymphocyte ratio; CALLY, C-reactive protein-albumin-lymphocyte index; HALP, Hemoglobin-albumin-lymphocyte-platelet score; NHR, Neutrophil-to-high-density lipoprotein cholesterol ratio; MHR, Monocyte-to-high-density lipoprotein cholesterol ratio; LHR, Lymphocyte-to-high-density lipoprotein cholesterol ratio; PHR, Platelet-to-high-density lipoprotein cholesterol ratio.

**Supplementary Table S5.** Benjamini–Hochberg false-discovery-rate sensitivity analysis of the standardized multivariable marker–outcome associations

| Variables | All-cause mortality_28-day_ | | | | All-cause mortality_1-year_ | | | |
| --- | --- | --- | --- | --- | --- | --- | --- | --- |
|  | HR | 95% CI | P value | q value | HR | 95% CI | P value | q value |
| Standardized SII | 1.157 | 1.048-1.277 | 0.004 | 0.024 | 1.109 | 1.005-1.224 | 0.039 | 0.088 |
| Standardized NLR | 1.141 | 1.029-1.265 | 0.012 | 0.043 | 1.091 | 0.978-1.217 | 0.118 | 0.224 |
| Standardized dNLR | 1.171 | 1.028–1.334 | 0.017 | 0.056 | 1.137 | 1.006–1.284 | 0.039 | 0.088 |
| Standardized MLR | 1.141 | 1.007–1.293 | 0.039 | 0.088 | 1.079 | 0.947–1.228 | 0.254 | 0.366 |
| Standardized NMLR | 1.142 | 1.030-1.267 | 0.012 | 0.043 | 1.092 | 0.978-1.218 | 0.118 | 0.224 |
| Standardized SIRI | 1.108 | 0.948-1.294 | 0.196 | 0.321 | 1.082 | 0.919-1.275 | 0.343 | 0.457 |
| Standardized PIV | 1.214 | 1.075-1.371 | 0.002 | 0.014 | 1.163 | 1.014-1.335 | 0.031 | 0.086 |
| Standardized PLR | 1.253 | 1.114-1.410 | <0.001 | 0.003 | 1.193 | 1.071-1.329 | 0.001 | 0.009 |
| Standardized NPR | 1.045 | 0.905-1.206 | 0.548 | 0.623 | 1.043 | 0.908-1.198 | 0.554 | 0.623 |
| Standardized PAR | 0.888 | 0.742-1.063 | 0.196 | 0.321 | 0.925 | 0.794-1.078 | 0.320 | 0.443 |
| Standardized CAR | 0.992 | 0.835-1.178 | 0.924 | 0.947 | 0.974 | 0.840-1.130 | 0.728 | 0.794 |
| Standardized CLR | 1.313 | 1.145-1.505 | <0.001 | 0.003 | 1.258 | 1.104-1.435 | <0.001 | 0.007 |
| Standardized CALLY | 0.990 | 0.808-1.212 | 0.921 | 0.947 | 1.005 | 0.874-1.155 | 0.947 | 0.947 |
| Standardized HALP | 1.046 | 0.922-1.186 | 0.483 | 0.580 | 1.069 | 0.955-1.198 | 0.247 | 0.366 |
| Standardized NHR | 0.870 | 0.748-1.012 | 0.072 | 0.152 | 0.946 | 0.833-1.073 | 0.385 | 0.495 |
| Standardized MHR | 0.905 | 0.775-1.058 | 0.211 | 0.330 | 0.945 | 0.821-1.087 | 0.426 | 0.529 |
| Standardized LHR | 0.759 | 0.626-0.920 | 0.005 | 0.026 | 0.819 | 0.703-0.954 | 0.010 | 0.043 |
| Standardized PHR | 0.814 | 0.677-0.978 | 0.028 | 0.084 | 0.902 | 0.777-1.047 | 0.177 | 0.319 |

The Benjamini–Hochberg procedure was applied jointly to the 36 nominal P values for the standardized associations of 18 markers with two mortality endpoints. A q value <0.05 was considered statistically significant after correction for multiple comparisons. Associations with P<0.05 but q≥0.05 were considered exploratory.

SII, Systemic immune-inflammation index; NLR, Neutrophil-to-lymphocyte ratio; dNLR, Derived neutrophil-to-lymphocyte ratio; MLR, Monocyte-to-lymphocyte ratio; NMLR, Neutrophil plus monocyte-to-lymphocyte ratio; SIRI, Systemic inflammation response index; PIV, Pan-immune-inflammation value; PLR, Platelet-to-lymphocyte ratio; NPR, Neutrophil-to-platelet ratio; PAR, Platelet-to-albumin ratio; CAR, C-reactive protein-to-albumin ratio; CLR, C-reactive protein-to-lymphocyte ratio; CALLY, C-reactive protein-albumin-lymphocyte index; HALP, Hemoglobin-albumin-lymphocyte-platelet score; NHR, Neutrophil-to-high-density lipoprotein cholesterol ratio; MHR, Monocyte-to-high-density lipoprotein cholesterol ratio; LHR, Lymphocyte-to-high-density lipoprotein cholesterol ratio; PHR, Platelet-to-high-density lipoprotein cholesterol ratio.

**Supplementary Table S6.** Multicollinearity diagnostics for the marker-specific multivariable Cox proportional hazards models

| Marker-specific model | Marker VIF | Marker tolerance | | Highest VIF in the model (predictor) | | Lowest tolerance in the model | |  |
| --- | --- | --- | --- | --- | --- | --- | --- | --- |
| **Panel A. Models for 28-day mortality (N = 396; events = 174)** | | | | | | | |  |
| SII | 1.219 | 0.821 | | 2.779 (SAPS II) | | 0.360 | |  |
| NLR | 1.269 | 0.788 | | 2.822 (SAPS II) | | 0.354 | |  |
| dNLR | 1.402 | 0.713 | | 2.761 (SAPS II) | | 0.362 | |  |
| MLR | 1.164 | 0.859 | | 2.831 (SAPS II) | | 0.353 | |  |
| NMLR | 1.267 | 0.790 | | 2.824 (SAPS II) | | 0.354 | |  |
| SIRI | 1.173 | 0.853 | | 2.831 (SAPS II) | | 0.353 | |  |
| PIV | 1.100 | 0.909 | | 2.758 (SAPS II) | | 0.363 | |  |
| PLR | 1.154 | 0.866 | | 2.716 (SAPS II) | | 0.368 | |  |
| NPR | 1.167 | 0.857 | | 2.743 (SAPS II) | | 0.365 | |  |
| PAR | 1.218 | 0.821 | | 2.704 (APACHE II) | | 0.370 | |  |
| CAR | 1.213 | 0.824 | | 2.711 (pH) | | 0.369 | |  |
| CLR | 1.383 | 0.723 | | 2.798 (APACHE II) | | 0.357 | |  |
| CALLY | 1.061 | 0.942 | | 2.705 (APACHE II) | | 0.370 | |  |
| HALP | 1.089 | 0.918 | | 2.721 (APACHE II) | | 0.368 | |  |
| NHR | 1.242 | 0.805 | | 2.714 (pH) | | 0.369 | |  |
| MHR | 1.217 | 0.821 | | 2.711 (pH) | | 0.369 | |  |
| LHR | 1.268 | 0.789 | | 2.561 (SAPS II) | | 0.390 | |  |
| PHR | 1.103 | 0.907 | | 2.662 (APACHE II) | | 0.376 | |  |
| **Panel B. Models for 1-year mortality (N = 396; events = 230)** | | | | | | | | |
| SII | 1.069 | | 0.936 | | 2.412 (APACHE II) | | 0.415 | |
| NLR | 1.171 | | 0.854 | | 2.415 (APACHE II) | | 0.414 | |
| dNLR | 1.278 | | 0.783 | | 2.392 (APACHE II) | | 0.418 | |
| MLR | 1.207 | | 0.829 | | 2.449 (APACHE II) | | 0.408 | |
| NMLR | 1.172 | | 0.853 | | 2.414 (APACHE II) | | 0.414 | |
| SIRI | 1.174 | | 0.852 | | 2.420 (APACHE II) | | 0.413 | |
| PIV | 1.077 | | 0.928 | | 2.404 (APACHE II) | | 0.416 | |
| PLR | 1.105 | | 0.905 | | 2.422 (APACHE II) | | 0.413 | |
| NPR | 1.153 | | 0.868 | | 2.451 (APACHE II) | | 0.408 | |
| PAR | 1.190 | | 0.840 | | 2.439 (APACHE II) | | 0.410 | |
| CAR | 1.186 | | 0.843 | | 2.425 (APACHE II) | | 0.412 | |
| CLR | 1.289 | | 0.776 | | 2.494 (APACHE II) | | 0.401 | |
| CALLY | 1.083 | | 0.923 | | 2.437 (APACHE II) | | 0.410 | |
| HALP | 1.060 | | 0.943 | | 2.451 (APACHE II) | | 0.408 | |
| NHR | 1.198 | | 0.835 | | 2.435 (APACHE II) | | 0.411 | |
| MHR | 1.137 | | 0.880 | | 2.432 (APACHE II) | | 0.411 | |
| LHR | 1.180 | | 0.847 | | 2.299 (APACHE II) | | 0.435 | |
| PHR | 1.082 | | 0.924 | | 2.425 (APACHE II) | | 0.412 | |

VIF, variance inflation factor. Tolerance was calculated as 1/VIF. Multicollinearity diagnostics were performed separately for each marker-specific multivariable Cox model using the same outcome-specific covariates as those reported in Table 2. Marker VIF and marker tolerance refer to the standardized inflammatory-immune marker included in the corresponding model. The highest VIF and lowest tolerance were calculated across all predictors included in each model. Potential multicollinearity was prespecified as VIF >=5 or tolerance <=0.20. No model met either criterion. For the 1-year CAR, CLR, and CALLY models, CRP was not included as an additional covariate because it is a component of these indices. Marker abbreviations are defined in Table 2.

**Supplementary Table S7.** Parsimonious multivariable Cox sensitivity analyses adjusted for age, sex, SOFA score, and lactate

| Marker | 28-day mortality (N/events = 396/174) | | | | 1-year mortality (N/events = 396/230) | | | |
| --- | --- | --- | --- | --- | --- | --- | --- | --- |
|  | Adjusted HR per 1-SD increase (95% CI) | P value | FDR q value | Marker PH P | Adjusted HR per 1-SD increase (95% CI) | P value | FDR q value | Marker PH P |
| SII | 1.161 (1.043-1.291) | 0.006 | 0.043 | 0.521 | 1.139 (1.022-1.269) | 0.018 | 0.070 | 0.669 |
| NLR | 1.141 (1.020-1.276) | 0.021 | 0.070 | 0.082 | 1.101 (0.981-1.235) | 0.101 | 0.243 | 0.615 |
| dNLR | 1.067 (0.932-1.221) | 0.347 | 0.553 | 0.070 | 1.011 (0.893-1.145) | 0.859 | 0.925 | 0.298 |
| MLR | 1.184 (1.038-1.351) | 0.012 | 0.055 | 0.595 | 1.142 (1.007-1.294) | 0.038 | 0.114 | 0.618 |
| NMLR | 1.143 (1.021-1.279) | 0.020 | 0.070 | 0.084 | 1.103 (0.983-1.238) | 0.096 | 0.243 | 0.616 |
| SIRI | 1.169 (1.003-1.364) | 0.046 | 0.127 | 0.422 | 1.099 (0.935-1.291) | 0.251 | 0.451 | 0.021 |
| PIV | 1.258 (1.109-1.427) | <0.001 | 0.004 | 0.703 | 1.209 (1.053-1.389) | 0.007 | 0.043 | 0.128 |
| PLR | 1.299 (1.151-1.464) | <0.001 | <0.001 | 0.200 | 1.277 (1.137-1.434) | <0.001 | <0.001 | 0.515 |
| NPR | 1.035 (0.896-1.195) | 0.643 | 0.747 | 0.935 | 1.011 (0.872-1.173) | 0.881 | 0.925 | 0.180 |
| PAR | 0.905 (0.773-1.060) | 0.216 | 0.410 | 0.983 | 0.954 (0.834-1.091) | 0.487 | 0.632 | 0.132 |
| CAR | 0.960 (0.816-1.129) | 0.619 | 0.743 | 0.187 | 0.911 (0.788-1.053) | 0.208 | 0.410 | 0.021 |
| CLR | 1.269 (1.112-1.447) | <0.001 | 0.004 | 0.626 | 1.193 (1.046-1.361) | 0.008 | 0.043 | 0.027 |
| CALLY | 0.945 (0.779-1.145) | 0.562 | 0.697 | 0.816 | 0.993 (0.877-1.125) | 0.913 | 0.925 | 0.465 |
| HALP | 1.052 (0.927-1.194) | 0.430 | 0.619 | 0.890 | 1.064 (0.949-1.192) | 0.287 | 0.492 | 0.749 |
| NHR | 0.933 (0.807-1.079) | 0.353 | 0.553 | 0.973 | 0.957 (0.843-1.085) | 0.492 | 0.632 | 0.812 |
| MHR | 0.990 (0.858-1.142) | 0.886 | 0.925 | 0.244 | 1.006 (0.885-1.143) | 0.925 | 0.925 | 0.680 |
| LHR | 0.870 (0.725-1.043) | 0.133 | 0.299 | 0.107 | 0.943 (0.819-1.086) | 0.419 | 0.619 | 0.505 |
| PHR | 0.882 (0.745-1.046) | 0.148 | 0.314 | 0.749 | 0.950 (0.829-1.089) | 0.461 | 0.632 | 0.106 |

Each standardized marker was evaluated separately in a Cox model adjusted for age, sex, SOFA score, and lactate. The Benjamini–Hochberg procedure was applied jointly to the 36 marker–outcome tests. The marker-specific proportional-hazards assumption was assessed using a marker × log(time) interaction term, with the corresponding P value reported as “Marker PH P”. A two-sided P<0.05 indicated evidence of a time-varying marker effect. Hazard ratios for SIRI, CAR, and CLR in the 1-year models represent overall summary associations averaged across follow-up and should not be interpreted as effects that remained constant over time.

**Supplementary Table S8.** Hospital-discharge landmark sensitivity analysis for 28-day and 1-year all-cause mortality among hospital survivors

| Variables | All-cause mortality_28-day_ | | | All-cause mortality_1-year_ | | |
| --- | --- | --- | --- | --- | --- | --- |
|  | HR | 95% CI | P value | HR | 95% CI | P value |
| Standardized SII | 1.096 | 0.958-1.254 | 0.183 | 1.106 | 0.981-1.247 | 0.098 |
| Standardized NLR | 1.084 | 0.935-1.256 | 0.287 | 1.110 | 0.973-1.265 | 0.120 |
| Standardized dNLR | 1.097 | 0.889-1.352 | 0.389 | 1.155 | 0.965–1.381 | 0.115 |
| Standardized MLR | 1.164 | 1.009-1.342 | 0.037 | 1.123 | 0.958–1.317 | 0.152 |
| Standardized NMLR | 1.086 | 0.937-1.259 | 0.275 | 1.111 | 0.974-1.267 | 0.117 |
| Standardized SIRI | 1.199 | 1.045-1.374 | 0.009 | 1.150 | 0.957-1.382 | 0.136 |
| Standardized PIV | 1.184 | 1.035-1.354 | 0.014 | 1.159 | 0.978-1.373 | 0.089 |
| Standardized PLR | 1.201 | 1.043-1.383 | 0.011 | 1.180 | 1.032-1.350 | 0.016 |
| Standardized NPR | 1.027 | 0.881-1.198 | 0.731 | 1.047 | 0.908-1.209 | 0.526 |
| Standardized PAR | 0.989 | 0.774-1.265 | 0.932 | 0.928 | 0.767-1.121 | 0.437 |
| Standardized CAR | 1.028 | 0.819-1.289 | 0.815 | 0.999 | 0.833-1.199 | 0.993 |
| Standardized CLR | 1.292 | 1.048-1.592 | 0.016 | 1.263 | 1.060-1.505 | 0.009 |
| Standardized CALLY | 0.921 | 0.544-1.558 | 0.758 | 0.830 | 0.528-1.304 | 0.419 |
| Standardized HALP | 1.033 | 0.903-1.180 | 0.637 | 1.083 | 0.964-1.217 | 0.178 |
| Standardized NHR | 0.932 | 0.752-1.155 | 0.521 | 0.975 | 0.823-1.154 | 0.767 |
| Standardized MHR | 1.114 | 0.895-1.386 | 0.333 | 0.988 | 0.815-1.196 | 0.898 |
| Standardized LHR | 0.880 | 0.679-1.141 | 0.335 | 0.872 | 0.729-1.044 | 0.135 |
| Standardized PHR | 0.941 | 0.746-1.187 | 0.608 | 0.919 | 0.771-1.096 | 0.347 |

Hospital discharge was used as the landmark at which eligible patients entered the risk set. Follow-up continued from hospital discharge until death or the corresponding administrative endpoint, while the underlying time scale and outcome definitions remained time since ICU admission: day 28 for the short-term analysis and day 365 for the 1-year analysis. In-hospital deaths did not enter this sensitivity analysis. The 1-year analysis included all 316 patients discharged alive, among whom 150 died after discharge. The 28-day analysis was restricted to patients discharged alive before day 28; 101 patients died after hospital discharge but within 28 days of ICU admission. Patients discharged on or after day 28 did not contribute risk time to the 28-day analysis.For all-cause mortality_28-day_, multivariate Cox regression analysis adjusted for age, heart rate, mean arterial pressure, ICU urine output_24h_, arterial oxygen saturation, PaCO_2_, Ph, lactate, serum potassium, PCT, BNP, ALT, Prothrombin time, D-dimer, SOFA_24h_, APACHE II_24h_, GCS_24h_, SAPS II score_24h_, SIRS_24h_, OASIS_24h_, Mechanical ventilation_48h_, CRRT_48h_.

For all-cause mortality_1-year_, multivariate Cox regression analysis adjusted for age, ICU urine output24h, PaCO_2_, arterial oxygen saturation, CRP, PCT, lactate, serum potassium, ALT, BNP, SOFA_24h_, APACHE II_24h_, GCS_24h_, SAPS II score_24h_, OASIS_24h_, and mechanical ventilation_48h_. Because CRP is a component of CAR, CLR, and CALLY, the multivariable models for these three indices excluded CRP as a covariate to avoid mathematical coupling. SII, Systemic immune-inflammation index; NLR, Neutrophil-to-lymphocyte ratio; dNLR, Derived neutrophil-to-lymphocyte ratio; MLR, Monocyte-to-lymphocyte ratio; NMLR, Neutrophil plus monocyte-to-lymphocyte ratio; SIRI, Systemic inflammation response index; PIV, Pan-immune-inflammation value; PLR, Platelet-to-lymphocyte ratio; NPR, Neutrophil-to-platelet ratio; PAR, Platelet-to-albumin ratio; CAR, C-reactive protein-to-albumin ratio; CLR, C-reactive protein-to-lymphocyte ratio; CALLY, C-reactive protein-albumin-lymphocyte index; HALP, Hemoglobin-albumin-lymphocyte-platelet score; NHR, Neutrophil-to-high-density lipoprotein cholesterol ratio; MHR, Monocyte-to-high-density lipoprotein cholesterol ratio; LHR, Lymphocyte-to-high-density lipoprotein cholesterol ratio; PHR, Platelet-to-high-density lipoprotein cholesterol ratio.

**Supplementary Table S9.** Multivariable Cox sensitivity analysis excluding the 48-h organ-support covariates included in the corresponding primary models

| Variables | All-cause mortality_28-day_ | | | All-cause mortality_1-year_ | | |
| --- | --- | --- | --- | --- | --- | --- |
|  | HR | 95% CI | P value | HR | 95% CI | P value |
| Standardized SII | 1.122 | 1.016-1.239 | 0.024 | 1.110 | 1.007-1.224 | 0.036 |
| Standardized NLR | 1.096 | 0.974-1.234 | 0.127 | 1.094 | 0.982-1.220 | 0.104 |
| Standardized dNLR | 1.126 | 0.968–1.309 | 0.125 | 1.146 | 1.003–1.309 | 0.045 |
| Standardized MLR | 1.110 | 0.967–1.274 | 0.139 | 1.073 | 0.942–1.223 | 0.289 |
| Standardized NMLR | 1.097 | 0.975-1.235 | 0.124 | 1.095 | 0.982-1.221 | 0.103 |
| Standardized SIRI | 1.097 | 0.937-1.284 | 0.248 | 1.078 | 0.916-1.269 | 0.364 |
| Standardized PIV | 1.209 | 1.068-1.368 | 0.003 | 1.165 | 1.015-1.338 | 0.030 |
| Standardized PLR | 1.273 | 1.134-1.428 | <0.001 | 1.197 | 1.075-1.332 | 0.001 |
| Standardized NPR | 1.032 | 0.893-1.193 | 0.669 | 1.036 | 0.901-1.191 | 0.619 |
| Standardized PAR | 0.908 | 0.759-1.086 | 0.290 | 0.938 | 0.805-1.092 | 0.408 |
| Standardized CAR | 0.999 | 0.840-1.187 | 0.991 | 0.977 | 0.842-1.134 | 0.762 |
| Standardized CLR | 1.288 | 1.126-1.473 | <0.001 | 1.261 | 1.108-1.436 | <0.001 |
| Standardized CALLY | 0.970 | 0.795-1.183 | 0.765 | 0.993 | 0.866-1.138 | 0.918 |
| Standardized HALP | 1.049 | 0.924-1.190 | 0.458 | 1.070 | 0.955-1.199 | 0.244 |
| Standardized NHR | 0.869 | 0.747-1.011 | 0.069 | 0.942 | 0.831-1.068 | 0.351 |
| Standardized MHR | 0.896 | 0.768-1.045 | 0.162 | 0.934 | 0.813-1.073 | 0.333 |
| Standardized LHR | 0.754 | 0.627-0.908 | 0.003 | 0.828 | 0.713-0.961 | 0.013 |
| Standardized PHR | 0.829 | 0.693-0.993 | 0.041 | 0.908 | 0.784-1.053 | 0.202 |

For all-cause mortality_28-day_, multivariate Cox regression analysis adjusted for age, heart rate, mean arterial pressure, ICU urine output_24h_, arterial oxygen saturation, PaCO_2_, Ph, lactate, serum potassium, PCT, BNP, ALT, Prothrombin time, D-dimer, SOFA_24h_, APACHE II_24h_, GCS_24h_, SAPS II score_24h_, SIRS_24h_, OASIS_24h_.

For all-cause mortality_1-year_, multivariate Cox regression analysis adjusted for age, ICU urine output_24h_, PaCO_2_, arterial oxygen saturation, CRP, PCT, lactate, serum potassium, ALT, BNP, SOFA_24h_, APACHE II_24h_, GCS_24h_, SAPS II score_24h_, OASIS_24h_. Because CRP is a component of CAR, CLR, and CALLY, the multivariable models for these three indices excluded CRP as a covariate to avoid mathematical coupling. SII, Systemic immune-inflammation index; NLR, Neutrophil-to-lymphocyte ratio; dNLR, Derived neutrophil-to-lymphocyte ratio; MLR, Monocyte-to-lymphocyte ratio; NMLR, Neutrophil plus monocyte-to-lymphocyte ratio; SIRI, Systemic inflammation response index; PIV, Pan-immune-inflammation value; PLR, Platelet-to-lymphocyte ratio; NPR, Neutrophil-to-platelet ratio; PAR, Platelet-to-albumin ratio; CAR, C-reactive protein-to-albumin ratio; CLR, C-reactive protein-to-lymphocyte ratio; CALLY, C-reactive protein-albumin-lymphocyte index; HALP, Hemoglobin-albumin-lymphocyte-platelet score; NHR, Neutrophil-to-high-density lipoprotein cholesterol ratio; MHR, Monocyte-to-high-density lipoprotein cholesterol ratio; LHR, Lymphocyte-to-high-density lipoprotein cholesterol ratio; PHR, Platelet-to-high-density lipoprotein cholesterol ratio.

**Supplementary Table S10.** Period-specific associations of SIRI, CAR, and CLR with 1-year all-cause mortality

| Marker | Days 1–90, adjusted HR per 1-SD increase (95% CI) | P value | Days 91–365, adjusted HR per 1-SD increase (95% CI) | P value |
| --- | --- | --- | --- | --- |
| SIRI | 1.143 (0.977–1.338) | 0.095 | 0.585 (0.247–1.386) | 0.223 |
| CAR | 0.920 (0.790–1.072) | 0.287 | 0.840 (0.549–1.286) | 0.422 |
| **CLR** | **1.222 (1.068–1.397)** | **0.003** | 0.876 (0.476–1.609) | 0.669 |

Each standardized marker was evaluated separately in a Cox model adjusted for age, sex, SOFA score, and lactate. Patients contributed risk time to each interval while remaining at risk. The division at 90 days was used as an exploratory aid to interpretation of the time-varying marker effects. Hazard ratios are expressed per 1-SD increase in the corresponding marker. P values were not adjusted for multiple comparisons. SIRI, systemic inflammation response index; CAR, C-reactive protein-to-albumin ratio; CLR, C-reactive protein-to-lymphocyte ratio; HR, hazard ratio; CI, confidence interval; SD, standard deviation.

**Supplementary Figure S1.** Time-varying associations of CAR, CLR, and SIRI with 1-year all-cause mortality

Adjusted time-varying hazard ratios associated with a 1-SD increase in CAR, CLR, and SIRI as a function of time since ICU admission. Estimates were obtained from extended Cox models including marker × log(time) interaction terms and adjusted for age, sex, SOFA score, and lactate. Solid blue curves represent the adjusted HR(t), and shaded areas represent the corresponding 95% CIs. The horizontal dashed line indicates HR=1, and the vertical dotted line indicates day 90. CAR, C-reactive protein-to-albumin ratio; CI, confidence interval; CLR, C-reactive protein-to-lymphocyte ratio; HR, hazard ratio; SD, standard deviation; SIRI, systemic inflammation response index; SOFA, Sequential Organ Failure Assessment.
